# Distinguishing Organomagnesium Species in the Grignard Addition to Ketones with X‐Ray Spectroscopy

**DOI:** 10.1002/chem.202402099

**Published:** 2024-11-07

**Authors:** Lorenzo Restaino, Riccardo Mincigrucci, Markus Kowalewski

**Affiliations:** ^1^ Department of Physics Stockholm University Albanova University Centre SE-106 91 Stockholm Sweden; ^2^ Elettra Sincrotrone Trieste SCpA Strada Statale 14 - km 163,5 in AREA Science Park 34149 Basovizza Trieste Italy

**Keywords:** X-ray absorption spectroscopy, Grignard reaction, Quantum Chemistry, X-ray photoelectron spectroscopy

## Abstract

The addition of Grignard reagents to ketones is a well‐established textbook reaction. However, a comprehensive understanding of its mechanism has only recently begun to emerge. X‐ray spectroscopy, because of its high selectivity and sensitivity, is the ideal tool for distinguishing between an ensemble of competing pathways. With this aim in mind, we investigated the concerted mechanism of the addition of methylmagnesium chloride (CH_3_MgCl) to acetone in tetrahydrofuran by simulating the X‐ray spectra of different molecules in solution. We used electronic structure methods to calculate the X‐ray absorption spectra at the Mg K‐ and L_1_‐edges and the X‐ray photoelectron spectra at the Mg K‐edge for different organomagnesium species, which coexist in solution due to the Schlenk equilibrium. The simulated spectra show that individual species can be distinguished throughout the different stages of the reaction. Each species has a distinct spectral feature which can be used as a fingerprint in solution. The absorption and photoelectron spectra consistently show a blue shift as the reaction progressed from reagents to products.

## Graphical abstract

We have calculated the X‐ray absorption and photoelectron spectroscopy of organomagnesium species in solution in the reaction of CH_3_MgCl with acetone. The spectra provide insight into the oxidation state of magnesium and the changes in the first solvation sphere.

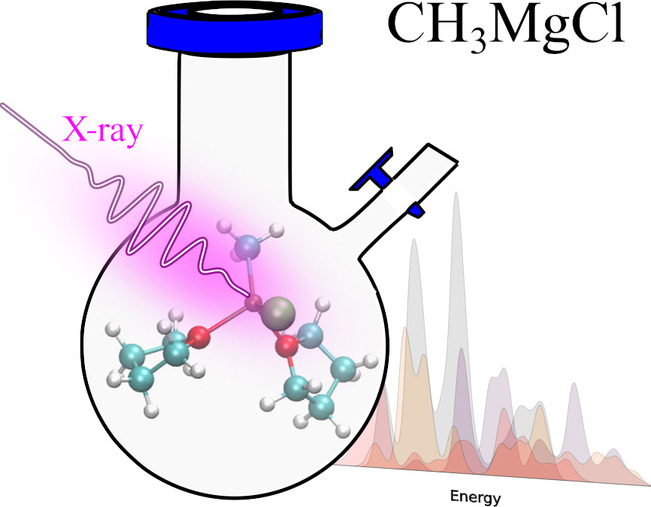

## Introduction

The Grignard reaction is an established chemical process that employs organomagnesium compounds to form new carbon‐carbon bonds. It was originally reported by Victor Grignard in 1900,^[1]^ who later received the Nobel Prize in 1912 for his discovery with Paul Sabatier. For more than a century, the Grignard reagent has been widely used as a coupling reagent for a variety of carbonyl derivatives, and its importance ranges from academia to industry.^[2]^ Despite its extensive use, the reaction mechanism is still not fully understood, as a result of the complex interplay of multiple factors. In fact, various organomagnesium species coexist in solution due to Schlenk equilibrium,[^3^, ^4^, ^5^, ^6^] leading to competing or parallel reaction pathways. Furthermore, the reaction mechanism is affected by the solvent, the nature of the substrate and the organometallic reagent.

Numerous attempts have been made to rationalize the reactivity spectrum.^[7]^ In summary, two types of mechanism have been identified at the two extremes of the spectrum: a polar or concerted mechanism (see the reaction of methylmagnesium bromide CH_3_MgBr with acetone^[8]^) and a radical or electron transfer mechanism (see the reaction of tert‐butylmagnesium chloride *t*‐C_4_H_9_MgCl with benzophenone^[9]^). The observed mechanism, whether electron transfer or concerted, depends on the reduction and oxidation potentials of both the substrates and the organomagnesium species involved in the reaction. The likelihood of a radical mechanism increases when substrates possess a low reduction potential, for example a molecule with a low‐lying $\pi^{*}$ system. In 2020, Peltzer et al. conducted a theoretical investigation of the reactivity of several organomagnesium species with acetaldehyde in tetrahydrofuran (THF).^[10]^ The authors explained how the properties of the substrate, the coordination of the Mg center, and the solvent dynamics affect the activation energy and determine the reaction pathways.

The structure and properties of Grignard reagents have been thoroughly studied via infrared (IR) spectroscopy,^[11]^ NMR[^12^, ^13^, ^14^, ^15^, ^16^, ^17^] and X‐ray crystallography.[^18^, ^19^, ^20^] ^25^Mg‐NMR, in particular, yields specific information about the magnesium coordination state when examining the Schlenk equilibrium. The species typically associated with the equilibrium have been recorded and identified through their distinct chemical shifts^[17]^ using ^25^Mg‐NMR.

The addition reaction of Grignard reagents to ketones has been investigated using ultraviolet (UV) and IR spectroscopy. Smith^[21]^ reported the emergence of two new bands in the UV spectrum when (CH_3_)_2_Mg or MgBr_2_ were added to certain aryl ketones in ethyl ether. Holm^[22]^ reported a change in the frequency of the carbonyl band in the IR spectrum when it coordinated magnesium.

X‐ray spectroscopy is an ideal tool for monitoring reactions involving changes in the coordination sphere of magnesium. Techniques like X‐ray absorption and photoelectron spectroscopy can probe the magnesium electronic structure and its local environment, even in complex mixtures. Indeed, it can monitor changes in the electron density of specific elements. These changes lead to an increase or decrease in electron affinity, which in turn shifts the absorption edge to higher or lower energies. Moreover, X‐ray spectroscopy can follow different types of bond rearrangements and charge transfer reactions, providing insight into geometric changes and, with sufficiently short pulses, the time scales of their elementary steps.^[23]^ In the context of organometallic reactions, the use of X‐ray spectroscopy to investigate the metallic center offers a number of significant advantages. First, the metal has a higher X‐ray cross‐section, resulting in stronger signals. Second, studying the metal is more informative than studying lighter elements, such as carbon, which are abundant and less distinctive. Third, the metal center is typically the catalytic site where changes in oxidation state and coordination environment occur, likely resulting in large difference spectra. The latter aspect is particularly relevant when using femtosecond pulses, which opens up the possibility for detailed time‐dependent studies. Developments^[24]^ in time‐resolved X‐ray spectroscopy are aimed at tracking ultra‐fast chemical rearrangements and charge transfers in molecules and materials,[^25^, ^26^] and to determine the reaction kinetics.[^23^, ^27^] The K‐shell absorption edge of Mg in solution is located around 1.3 keV. In recent decades, only a few X‐ray studies have been conducted on Grignard reagents in ether,[^28^, ^29^, ^30^, ^31^, ^32^] due to the difficulty in performing experiments in liquid under high‐vacuum conditions. Recent technological advances^[33]^ enable experiments to be carried out at the Mg K‐edge in large‐scale facilities. Experiments at the Mg K‐edge have increased due to interest in organomagnesium electrolytes for batteries.^[34]^ Only a few X‐ray photoelectron spectroscopic (XPS) investigations have been conducted on Grignard species at the Mg K‐edge, with a primary focus on surface characterization rather than investigating addition reactions.[^35^, ^36^]

**Figure 1:**
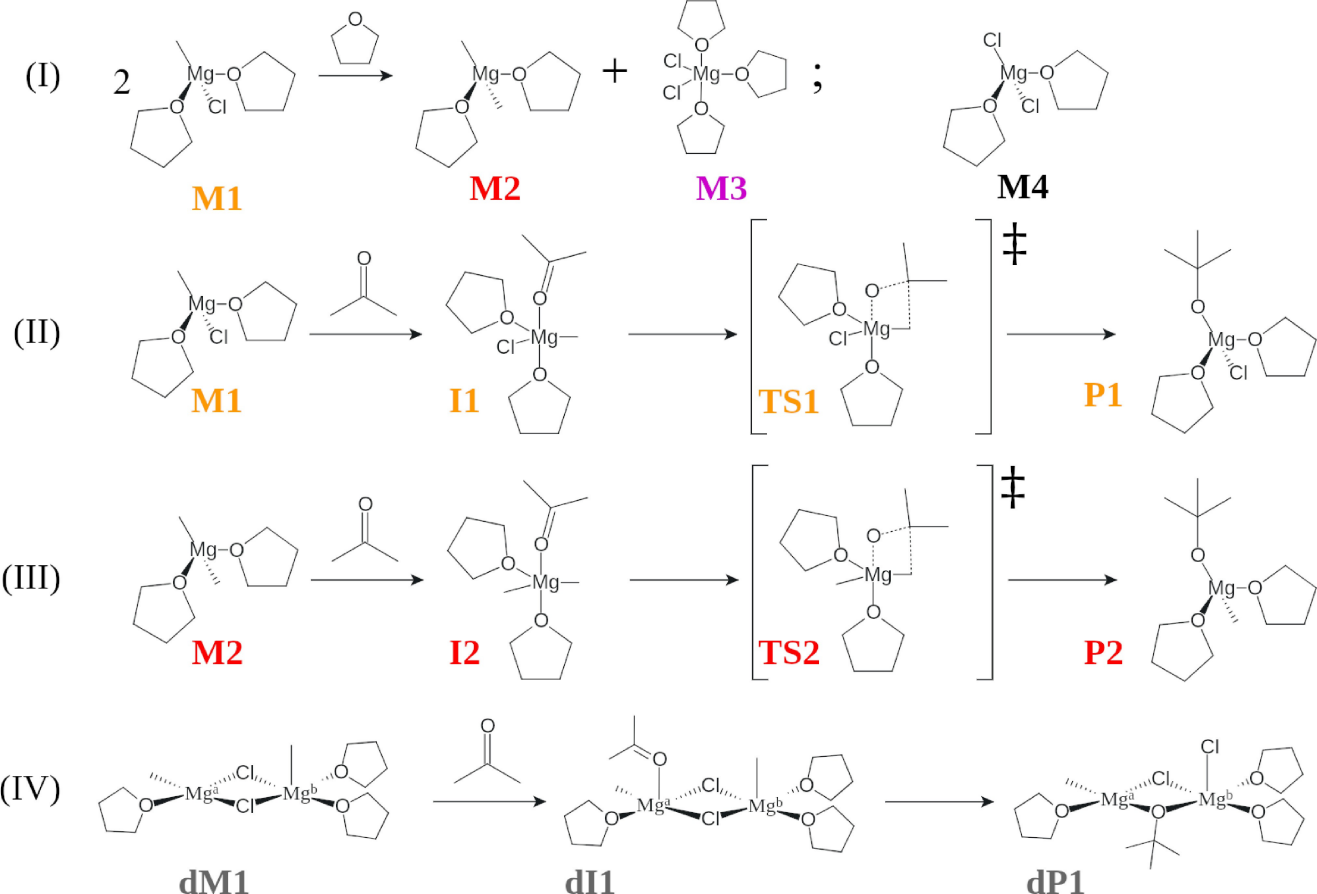
Structures and labels of the organomagnesium species associated with the reaction of CH_3_MgCl with acetone in THF; the color scheme introduced in the labels will be used consistently throughout the paper. (I): Species in solution due to Schlenk equilibrium; (II) and (III): Schematics of the pathway towards carbon‐carbon bond formation, following two distinct tetra‐coordinated organomagnesium species; (IV): Schematics of carbon‐carbon bond formation for the dinuclear complex with the lowest activation energy, as calculated by Peltzer et al.^[10]^ M indicates starting structures, I indicates intermediates of reaction, TS transition states and P products. Labels preceded by the letter “d” indicate dimers.

Following Peltzer's findings, in this work we focused our attention on those species characterized by the lowest activation energy toward the concerted mechanism, in the reaction of methylmagnesium chloride (CH_3_MgCl) with acetone in THF. The equilibria and the molecules associated with the reaction are reported and labeled in Figure 1.

We employ simulations of time‐independent X‐ray absorption and time‐independent X‐ray photoelectron spectroscopy to theoretically investigate potential reaction pathways and identify different species in solution. For this purpose, we characterized minima structures, like reagents, intermediates of reaction, and products, and simulated their X‐ray spectra using quantum chemistry calculations. Specifically, we monitored the Mg K‐edge and L_1_‐edge which allow us to gain insight into the changes occurring in the first coordination sphere of Mg throughout the reaction. This information provides crucial details on the geometric transformations that take place during the process. In this paper, we present a static picture of the reaction, which represents the first step towards a more comprehensive and detailed time‐resolved picture.

## Results and Discussion

### Molecular Structures

Most of the reported crystal structures of Grignard compounds are four‐coordinated and have a distorted tetrahedral geometry.^[18]^ The monomeric complex has the structure RMgXL_2_ where R is the organic group, X is the halide and L is the solvent (here THF). Binuclear structures can also exist in THF and diethyl ether solution, and the ratio between monomers and dimers is governed by the Schlenk equilibrium.[^8^, ^37^] In these dinuclear structures, two tetrahedral Mg centers are commonly bridged by two halogen atoms. Nevertheless, the structure of Grignard reagents in solution may differ from that of the crystalline solid due to lattice packing effects. The molecular structures investigated in this work are reported and labeled in Figure 1.

The Schlenk equilibrium (see Figure 1(I)) generates two new species: (CH_3_)_2_MgL_2_ (M2) and MgCl_2_L_x_. Calculations^[6]^ showed that the most likely solvation state for MgCl_2_L_x_ is with x=3 (M3), arranged in trigonal‐bipyramidal coordination at Mg with both chlorine atoms occupying equatorial positions. In the ground‐state geometry of CH_3_MgClTHF_2_ (M1) and (CH_3_)_2_MgTHF_2_ (M2), the Mg−C_Me_ (Me=methyl group) distance is approximately the same at 2.1 Å. The average bond length between the metal center and the oxygen atom of the solvent molecules, (Mg−O_THF_), is ~2.1 Å for both structures. The Mg−Cl distance in M1 is the longest bond length, with a value of 2.38 Å. Both M1 and M2 show distortions from the ideal tetrahedral geometry with bond angles of 109.96° and 131.49°, and 105.2° and 135.98° respectively (compared to the ideal value of 109.5°).

To form the new C−C bond, the carbonyl substrate (here acetone) must first coordinate the magnesium atom. The coordination of the acetone molecule leads to the formation of a triangular bipyramidal (TBP) structure. The optimized minima, confirmed by frequency analysis, precede the transition state and thus are intermediates of reaction (see I1 and I2). The acetone occupies one of the apical sites, while a solvent molecule, the halogen atom (if present) and the R group of the Grignard reagent occupy equatorial sites. At the transition state, the Mg, CH_3_ and CO all lie in the same plane (dihedral angle $\theta <10^{\circ }$ ). This TBP structure is in agreement with the calculations of Peltzer et al.^[10]^ In TS2, the carbon with the longest Mg−C_Me_ bond forms the new bond with the carbonyl carbon.

The products of the concerted reaction, P1 and P2, have a distorted tetrahedral geometry and are characterized by the presence of the newly formed carbon‐carbon bond. These species are precursors of the final products of the Grignard reaction, which are then obtained by hydrolysis. The calculations in THF predict the new carbon‐carbon bond, C_Me_−C_ACE_, to be 1.5 Å for both P1 and P2. Although dimer formation is essential for the evolution of the Schlenk equilibrium reaction, experiments^[38]^ and calculations^[6]^ have shown that such dinuclear structures (dM1) are present only in small concentrations in THF.

Before discussing the spectra, we must stress that a direct comparison between the core‐excitation energies of the X‐ray absorption spectra and the calculated binding energies of the X‐ray photoelectron spectra is not directly possible. Different methods have been used to describe the core‐hole states in XAS and XPS. Nonetheless, we can compare the relative energy shifts between species and discuss the general trend in energy as the reaction progresses from reactants (M1, M2 or dM1) to products (P1, P2 or dP1).

### X‐ray Absorption Spectra at Mg K‐edge

**Figure 2:**
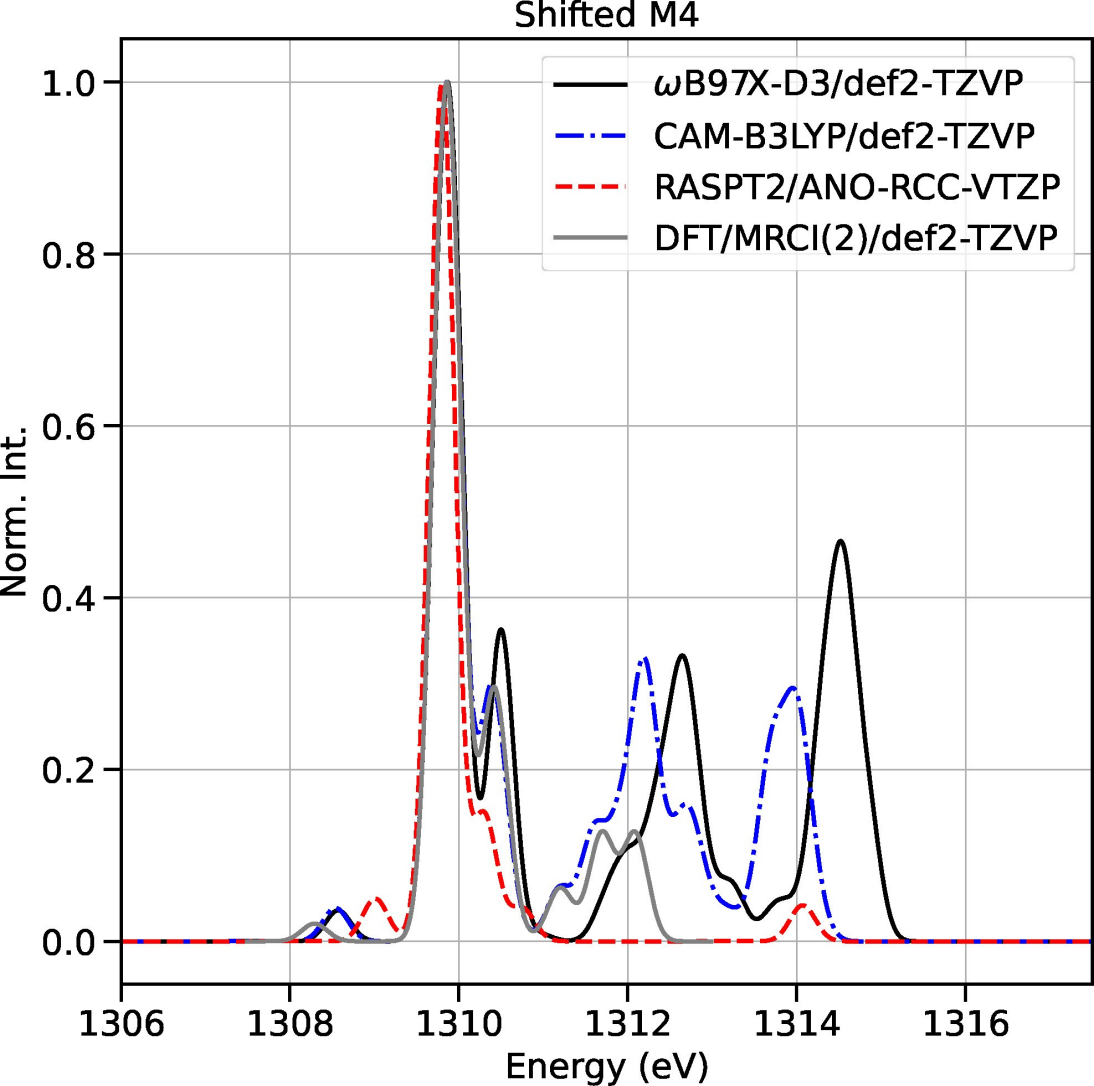
Comparison between XAS of M4 at Mg K‐edge: *ω*B97X‐D3 (solid, black), CAM‐B3LYP (dashdot, blue), XMS‐RASPT2 (dashed, red) and DFT/MRCI(2) (solid, gray). The spectra were shifted according to the alignment scheme. Spectra are normalized with respect to their maximum intensity to compare different methods.

We now discuss the calculated X‐ray absorption spectra at the Mg K‐edge of the optimized minima presented in the previous section. The energies of the Mg K‐edge XAS spectra are shifted using the NEXAFS spectrum of MgCl_2_THF_2_ (M4) as a reference (see Computational Details). In Figure 2, we compare the XAS spectra of M4 simulated with different methods (LR‐DFT, XMS‐RASPT2 and DFT/MRCI(2)). The *ω*B97X‐D3 (black, solid line) and CAM‐B3LYP (green, dashdotted line) functionals predicted spectra with very similar shapes, differentiated by a small offset in the energy axis. The XMS‐RASPT2 method (red, dashed line) accurately reproduced the first strong peak and the shoulder to its right. However, multi‐reference calculations for large molecules with a large number of roots are a challenging task, because of the high computational cost, convergence issues and a strong dependence on the choice of the active space. To provide further benchmarking of the LR‐DFT calculations, the absorption spectrum of M4 was also calculated using the DFT/MRCI(2) framework (more in the Computational Details section). Despite the significantly reduced computational cost of DFT/MRCI(2) in comparison to XMS‐RASPT2, the spectrum (grey line, Figure 2) accurately reproduces the most prominent peaks within the 1308–1313 eV energy window. The molecular orbital plots of the active spaces employed in the XMS‐RASPT2 and DFT/MRCI(2) calculations are displayed in the Supplementary Information (SI) in Figures S7 and S9.

**Figure 3:**
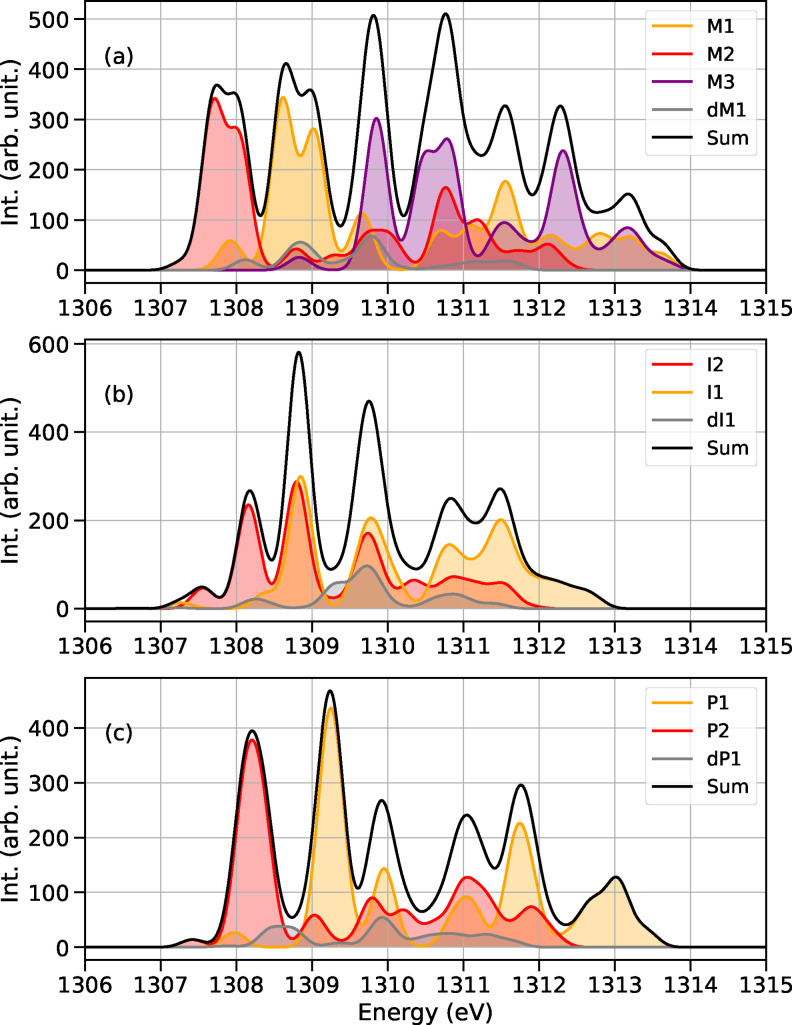
Sum of absorption spectra at the Mg K‐edge of organomagnesium species coexisting in THF at different stages of the reaction. (a) Sum of the spectra of the species involved in the Schlenk equilibrium; (b) sum of the spectra of intermediates of the reaction; (c) sum of the spectra of products with the newly formed carbon‐carbon bond.

Monomeric species, such as M1 (RMgXL_2_), M2 (R_2_MgL_2_), and M3 (MgX_2_L_3_), as well as the binuclear dM1, can co‐exist in the solution. Consequently, the X‐ray absorption spectrum measured in THF will result from the contributions of each species, with their respective signals weighted by their molar fractions. The species M4, used to benchmark excitation energies, is in equilibrium with MgCl_2_THF_3_ (M3) at a ratio of 0.01 : 1.0, and therefore it is unlikely to have a significant impact on the actual spectrum in a solution. We show the cumulative spectrum in solution in Figure 3, with the assumption of equal molar fraction for all monomeric species. We do not include the spectrum of the tetracoordinated M4, since the ratio between M4 and M3 is heavily skewed towards M3. Binuclear species in THF are known to be present at very low concentrations.^[38]^ To account for the low concentration in the spectra, their contribution are weighted with 0.1 to give a more realistic representation of the spectrum in solution. A comparison between the TDA and XMS‐RASPT2 spectra of M1 is available in the SI in Figure S6, and additional absorption spectra showing the evolution of the signals at different stages of the reaction, can be found in Figs. S10, S11 and S12. X‐ray absorption probes transitions to core‐excited states. At the Frank‐Condon point, such transitions can be identified as excitations from core orbitals to singly occupied molecular orbitals (SOMOs) or lowest unoccupied molecular orbitals (LUMOs). The most prominent peaks appearing in Figure 3 have been identified and assigned to a dominant, characteristic orbital excitation. The assignment is reported in Table 1, and the corresponding natural transition orbitals are available in the SI in figures S1 to S4. According to the calculations, the absorption spectra of M1 (Figure 3(a), orange curve) and M2 (red curve) present two closely spaced, strong peaks at energies between 1308 and 1309 eV. The pair of signals is separated by approximately 0.4 eV in M1 and by 0.3 eV in M2. In both cases, their intensity ratio is 0.8 : 1. A first low‐intensity feature is present in the absorption spectrum of each organomagnesium species, and it is observed at low photon energy preceding a stronger peak. As reported in Table 1, this weak feature has been attributed to a transition from the Mg 1s to a $\sigma^{*}$ involving the Mg atom and the carbon of the methyl group, with contributions from the other ligands. The transition corresponding to the excitation from the Mg 1s to an essentially low‐lying empty p orbital of Mg (Table 1, peak 2) undergoes the most significant energy change. The different environments surrounding the Mg atom exert a considerable influence on this p orbital, which shows a shift of approximately 1 eV between M1 and M3. By following this peak, M1, M2 and M3 can be readily distinguished. The X‐ray absorption spectrum of dM1 is characterized by three well‐separated features at 1308.1, 1308.8 and 1309.8 eV with an intensity ratio of 0.3:0.8:1 and a cluster of low intensity signals occurring at higher photon energies (>1310.5 eV). The chemical environment surrounding the two metal centres in the dimer is sufficiently diverse to separate the two Mg 1s to p orbital transition (peaks 3 and 6 in Table 1), with an energy separation of 1 eV between Mg^*a*^ and Mg^*b*^ (see Figure 1 for labelling). Figure S12(a) of the SI offers a better visual representation of the dimer spectrum. It should be noted that the absorption peaks of the binuclear structure dM1 overlap with the primary peaks of M1 and M3, occurring at approximately 1309 and 1310 eV, respectively. This observation is expected due to the similar chemical environment around the Mg atom between the dimer and the M1 and M3 monomers.

**Table 1:** Assignment of selected absorption peaks in the K‐edge spectra. Energies (ħ*ω*) in eVs. Numbers in the assignment column refer to the corresponding natural transition orbitals, displayed in the SI in Figs. S1 to S4. THF denotes tetrahydrofuran, and Me denotes the methyl group.

Reactants			Intermediates			Products		
	ℏω	Assignment		ℏω	Assignment		ℏω	Assignment
M1	1307.9	1, $\sigma_{\mathrm{Mg}-\mathrm{Me}}^{*}$	I1	1308.3	1, $\sigma_{\mathrm{Mg}-\mathrm{Me}}^{*}$	P1	1307.9	1, $\sigma_{\mathrm{Mg}-\mathrm{Me}}^{*}$
	1308.6	2, Mg p		1308.8	2, Mg p		1309.2	2, Mg p
	1309.0	3, Mg p + $\sigma_{\mathrm{Mg}-O}^{*}$		1309.6	3, Mg p + $\sigma_{\mathrm{Mg}-O}^{*}$		1309.3	3, Mg p + $\sigma_{\mathrm{Mg}-O}^{*}$
	1311.5	4, Mg p + $\sigma_{\mathrm{Mg}-\mathrm{Cl}}^{*}$		1310.0	4, $\sigma_{\mathrm{Mg}-O}^{*}$		1309.9	4, $\sigma_{\mathrm{Mg}-O}^{*}$
M2	1307.2	1, $\sigma_{\mathrm{Mg}-\mathrm{Me}}^{*}$	I2	1307.5	1, $\sigma_{\mathrm{Mg}-\mathrm{Me}}^{*}$	P2	1307.4	1, $\sigma_{\mathrm{Mg}-\mathrm{Me}}^{*}$
	1307.7	2, Mg p		1308.1	2, Mg p + $\sigma_{\mathrm{Mg}-\mathrm{THF}}^{*}$		1308.1	2, Mg p
	1308.0	3, Mg p + $\sigma_{\mathrm{Mg}-O}^{*}$		1308.7	3, Mg p + $\sigma_{\mathrm{Mg}-O}^{*}$		1308.3	3, Mg p + $\sigma_{\mathrm{Mg}-O}^{*}$
	1310.7	4, Mg p		1308.8	4, Mg p + $\sigma_{\mathrm{Mg}-O}^{*}$		1309.0	4, $\sigma_{\mathrm{Mg}-O}^{*}$
dM1	1308.1	1, $\sigma_{{\mathrm{Mg}}^{a}}^{*}$	dI1	1308.2	1, $\sigma_{{\mathrm{Mg}}^{b}}^{*}$	dP1	1307.8	1, $\sigma_{{\mathrm{Mg}}^{a}}^{*}$
	1308.1	2, $\sigma_{{\mathrm{Mg}}^{b}}^{*}$		1309.2	2, Mg^*b*^ p		1308.4	2, Mg^*a*^ p
	1308.7	3, Mg^*a*^ p		1309.3	3, Mg^*a*^ p		1308.7	3, Mg^*a*^ p
	1308.9	4, Mg^*a*^ p		1309.6	4, Mg^*b*^ p + $\sigma_{{\mathrm{Mg}}^{b}-\mathrm{THF}}^{*}$		1309.3	4, Mg^*a*^ p + $\sigma_{{\mathrm{Mg}}^{a}-\mathrm{Cl}}^{*}$
	1309.4	5, Mg^*b*^ p + $\sigma_{{\mathrm{Mg}}^{b}-\mathrm{Me}}^{*}$		1309.6	5, Mg^*b*^ p		1309.8	4, Mg^*b*^ p + $\sigma_{{\mathrm{Mg}}^{b}-\mathrm{Cl}}^{*}$
	1309.7	6, Mg^*b*^ p		1309.7	6, Mg^*a*^ p		1310.0	6, Mg^*b*^ p
	1309.8	7, Mg^*b*^ p		1309.8	7, $\sigma_{{\mathrm{Mg}}^{b}}^{*}$		1310.8	7, $\sigma_{{\mathrm{Mg}}^{b}}^{*}$
M3	1308.8	1, $\sigma_{\mathrm{Mg}-\mathrm{Cl}}^{*}$						
	1309.8	2, Mg p						
	1310.4	3, Mg p + $\sigma_{\mathrm{Mg}-O}^{*}$						
	1310.8	4, Mg p						
M4	1308.5	1, $\sigma_{\mathrm{Mg}-\mathrm{Cl}}^{*}$						
	1309.8	2, Mg p						
	1309.9	3, Mg p + $\sigma_{\mathrm{Mg}-O}^{*}$						
	1310.5	4, Mg p + $\sigma_{\mathrm{Mg}-\mathrm{Cl}}^{*}$						

The reaction intermediates (I1, I2, dI1) depicted in Figure 1 have a distorted triangular bipyramidal geometry, with the oxygen of the acetone weakly coordinating the Mg center. As mentioned above, this structure undergoes a rearrangement in the transition state to allow the concerted four‐point reaction. The spectra of the reaction intermediates are shown in Figure 3(b). The two strong peaks (with a 1:0.8 intensity ratio) of M1 vanish, and a single peak at 1308.8 eV appears in I1. The differences between M2 and I2 are more pronounced and the two species are easily distinguishable from each other due to an overall blue shift of the XAS spectrum of nearly 1 eV. The shape of the XAS spectrum of the dimer is not significantly affected by the presence of an acetone ligand. The spectrum of dI1 is approximately blue shift of 0.3 eV with respect to dM1. Furthermore, the ratio between the first three signals changes to 0.3:0.5:1. See Figure S12(b) of the SI for an easier comparison.

The XAS spectra of the reaction products are shown in panel (c) of Figure 3. P1 and P2 have XAS spectra that are both characterized by a single strong peak following the low intensity feature (Mg $\sigma^{*}$ ). Similarly to the case of reactants, a considerable energy shift of the Mg 1s→Mg p excitation can also be observed in the case of products. In fact, this transition, occurring at 1309.2 and 1308.1 eV, respectively, for P1 and P2, is red‐shifted by 1 eV, making it easy to distinguish between the two species. The XAS spectrum of dP1 is significantly different from I1, reflecting the different chemical environments surrounding the two Mg centers in the reaction product. The same pattern observed in the reactants is also visible for the products, where a clear energy separation of the transitions from the two metal centers is evident. The spectrum can be divided into two parts: at lower photon energies (between 1307 and 1309.5 eV) the peaks are due to one‐electron transitions from the 1s orbital of the magnesium atom labeled Mg^*b*^, while at higher photon energies (>1309.5 eV) the transitions involve the 1s electron of Mg^*a*^ (see also Figure S12(c) of the SI).

In summary, reaction pathways (II) and (III) result in a blue shift over the course of the reaction of 0.6 and 0.5 eV, respectively. In both cases, the reactants and products of each pathway show clearly different signatures. The dimer case is complex, showing distinct excitations from the two magnesium centers. However, these excitations may also overlap with those of the monomeric species.

### X‐ray Absorption Spectra at Mg L_1_‐edge

**Figure 4:**
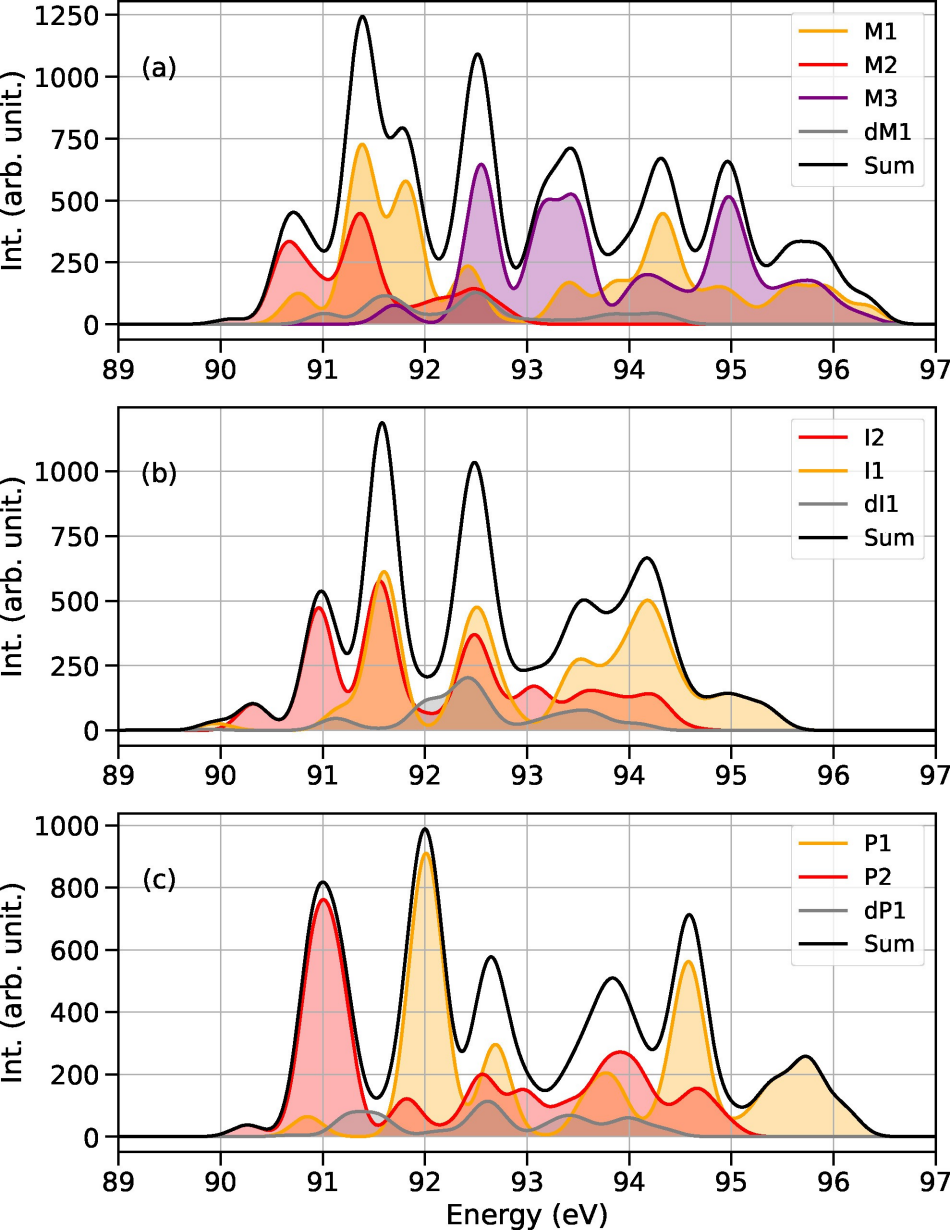
Sum of absorption spectra at the Mg L_1_‐edge of organomagnesium species coexisting in THF at different stages of the reaction. (a) Sum of spectra of the species involved in the Schlenk equilibrium; (b) sum of spectra of intermediates of reaction; (c) sum of spectra of the reaction products with the newly formed carbon‐carbon bond.

We now move to the analysis of the Mg L_1_‐edge, reported in Figure 4. We found that the K‐edge and L_1_‐edge spectra have many similarities in shape. The most striking differences can be found in the absorption spectrum for the reactant species M1, M2, M3 and dM1 in panel (a). The two transitions obey the same selection rule, because the electron is promoted from total symmetric orbitals (Mg 1s and Mg 2s), but the energy required for the vertical transition is, of course, different.

The results and observations of the K‐edge are also applicable to the L_1_‐edge case. We observe a blue shift of 0.6 eV for pathways (II) and 0.5 eV (III). Furthermore, the most prominent peaks in the collective spectrum in THF can be clearly attributed to different organomagnesium compounds. Thus, by studying the reaction at the L_1_‐edge, we maintain the selectivity of the K‐edge, while also gaining access to an energy range that may be more readily accessible in experiments. However, it is important to note that the Mg L_1_‐edge may present an experimental challenge due to the considerable carbon and oxygen background caused by the solvent. To provide an estimate: for a 1 molar solution of Grignard reagent in THF, we estimated the change in transmission at 89.4 eV due to Mg to be approximately 2 % or less of the total transmission.[^39^, ^40^] In contrast, the estimated change in transmission at the Mg K‐edge is 86 % of the total transmission under the same conditions.

### X‐ray Photoelectron Spectra

Before discussing the results of the calculations and discussing the Mg 1s binding energies (BEs), we can predict the trend in the chemical shift by analyzing at the chemical environment of the Mg atoms. The initial observation is that THF acts as a weak electron‐donating ligand, thus shielding the charge on the magnesium atom. Furthermore, the Mg−O distance is approximately 2.1 Å for all the species studied, therefore we can assume that the screening effect of THF is uniform for molecules with the same solvation state. M4 and the binuclear structure likely have the highest Mg 1s ionization potentials due to the strong withdrawal of electron charge from the two electronegative chlorine ligands. The Mg atom in M2 is expected to have the smallest BE due to the two methyl ligands, acting as electron‐donating groups and screening the Mg charge. The species M1 represents an intermediate situation with one methyl group and one chlorine atom. In binuclear structures, the two magnesium centers, Mg^*a*^ and Mg^*b*^, share most ligands, suggesting similar binding energies.

We calculated the binding energies (BEs) of Mg 1s using the ΔK‐S method with three different functionals (B3LYP, M06 and M06‐2X). Moreover, we estimated the energy shift due to the stabilizing effect of the solvent by comparing the results with and without PCM corrections. We found that the THF solvent stabilizes the core‐ionized cations by 4–5 eV, thereby lowering the BEs of the organomagnesium molecules in solution.

**Figure 5:**
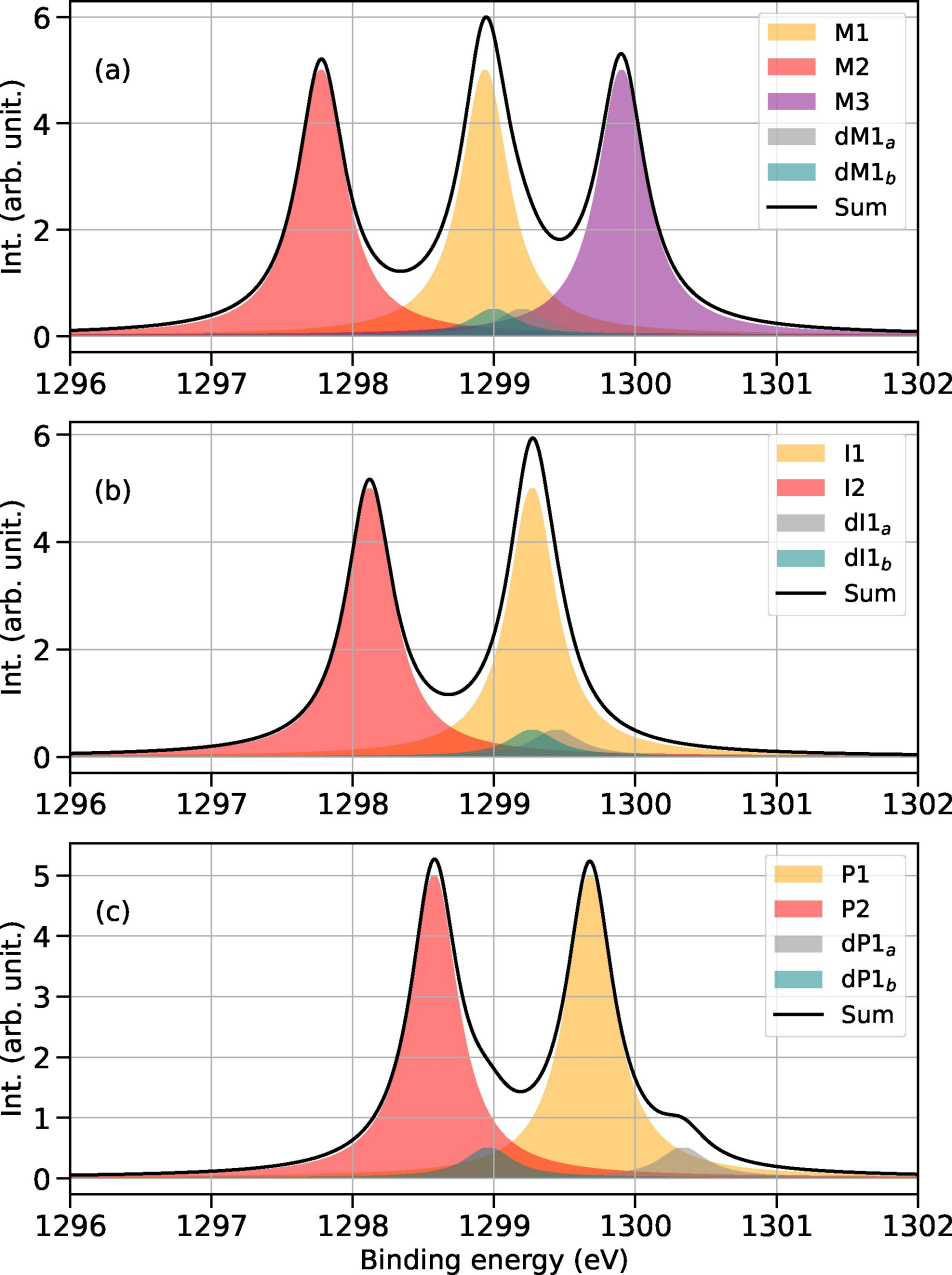
Sum of X‐ray photoelectron spectra at Mg K‐edge of Grignard species coexisting in solution, calculated with the ΔK‐S method at the B3LYP/ANO‐RCC‐VTZP level of theory.

The ΔK‐S calculations present a chemically intuitive picture, as shown in Figure 5. They suggest that the oxidation state of the Mg atoms is in the following order: dP1 (Mg^*a*^)>M3>P1≈dI1 (Mg^*a*^)≈dI1 (Mg^*b*^)>I1≈dM1 (Mg^*a*^)>dM1 (Mg^*b*^)≈dP1 (Mg^*b*^)>M1>P2>I2>M2. This order is in agreement with the predictions that were made solely on the basis of the chemical arguments discussed above. The three functionals predict similar absolute energies and reproduce the same trend as the reaction progresses from reagents to products. Therefore, we limit our discussion to B3LYP, but report all the calculated energies in Table 2.

**Table 2:** Binding energies (eV) for different Grignard species calculated with the ΔKohn‐Sham method.

Species	B3LYP	M06	M06‐2X
M1	1298.94	1298.97	1298.86
I1	1299.27	1299.31	1299.21
P1	1299.68	1299.74	1299.58
M2	1297.78	1297.83	1297.72
I2	1298.12	1298.16	1298.08
P2	1298.58	1298.66	1298.50
dM1 (Mg^*a*^)	1299.20	1299.28	1299.17
dM1 (Mg^*b*^)	1299.00	1299.06	1298.97
dI1 (Mg^*a*^)	1299.44	1299.52	1299.42
dI1 (Mg^*b*^)	1299.27	1299.35	1299.26
dP1 (Mg^*a*^)	1300.75	1300.42	1300.28
dP1 (Mg^*b*^)	1298.96	1299.04	1298.93
M3	1299.90	1299.95	1299.82

The sum of X‐ray photoelectron spectra of various contributing species in solution is shown in Figure 5. The identification of species M2 and M3 in panel (a) of Figure 5 is straightforward: the estimated binding energies are 2.3 eV apart and easily distinguishable. However, the BEs for M1 and dM1 are close together, with only a 0.2 eV difference. Furthermore, it would be difficult to differentiate between the two magnesium centers of the binuclear structure since they are only 0.2 eV apart. A similar situation is observed for the intermediates of reaction in panel (b). The I2 species is well separated from the other intermediates, while I1, dI1^*a*^, and dI1^*b*^ are 0.2 eV apart. However, all Mg centers in the product species are easily distinguishable. The binding energy of P2 is 1 eV and 0.4 eV lower than P1 and dP1^*b*^, respectively. Additionally, the binding energies of the magnesium atoms in the dimer product are shifted by almost 2 eV, which makes it possible to assign the peak without any confusion. This finding confirms the results of the XAS spectrum analysis of dP1, which showed a clear distinction between the two magnesium centers and their chemical environment. Overall, the XPS spectra calculated with the ΔK‐S method show a minimum blue shift of 0.75 eV from the initial species (M1, M2, dM1) to the products (P1, P2, and dP1) along the reaction paths (I), (II) and (IV), which is consistent with what has been observed in the XAS spectra.

**Figure 6:**
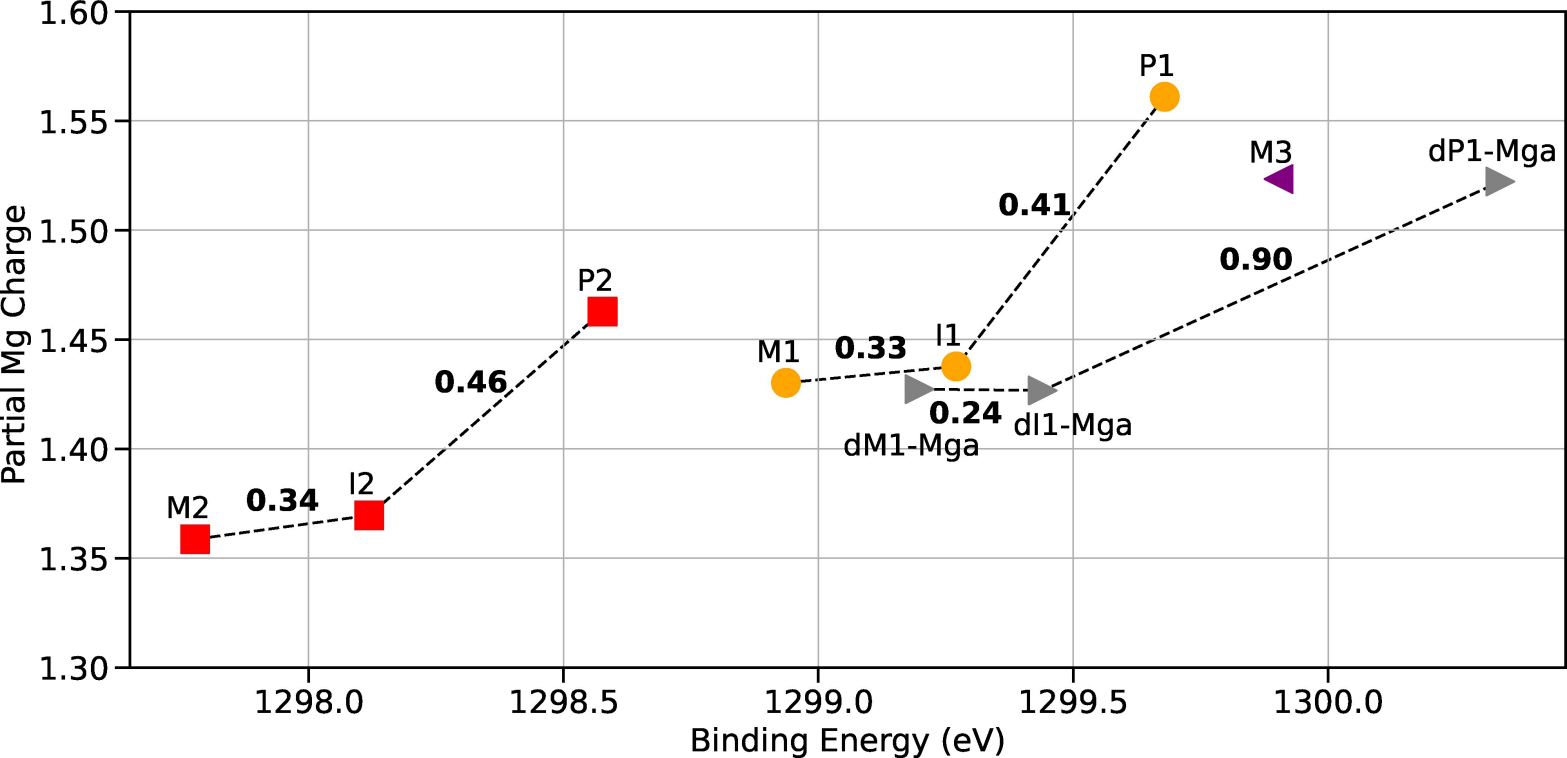
Correlation between the ΔK‐S/B3LYP binding energies (eV) of Mg 1s electron and the local partial charge on magnesium for different species in solution. Dashed lines indicate ΔBEs reported in bold.

The correlation between the localized charge of the probed atom and the binding energy, which should reflect the chemical shift (CS), calculated using the LoProP approach, is shown in Figure 6. An increase in the ionization potential generally coincides with an increase in the partial charge of magnesium, indicating a higher oxidation state of the atom. The change in the partial charge of the metal is more pronounced between the intermediates and the final product than between the reactants and intermediates. We attribute the increase in binding energy from reactants to intermediates to a change in the geometry of the complex (from a distorted tetrahedron to a triangular bipyramid) rather than a significant change in the oxidation state of the metal. However, the significant variation of the partial atomic charge of Mg observed in the products (Δq≈0.1 ) strongly suggests a change in the oxidation state. This change is most likely due to the presence of the newly formed *t*‐butoxy ligand, which acts as a strong base. The observed pattern is reproduced for all the reaction pathways investigated. Additional correlation plots calculated with M06 and M06‐2X are available in the SI.

## Conclusions

We theoretically studied the reaction of methylmagnesium chloride with acetone in THF using X‐ray spectroscopy. We selected mono‐ and bi‐nuclear magnesium structures present in solution due to Schlenk equilibrium and with the lowest activation energy toward the concerted mechanism. We employed LR‐DFT to calculate their X‐ray absorption spectra at the Mg K‐edge and L_1_‐edge, and the ΔKohn‐Sham method to compute the Mg 1s binding energies. We found that, as the reaction progresses towards the products, the absorption peak corresponding to the Mg 1s to low‐lying unoccupied Mg p orbital experiences a blue shift of 0.6 eV for the reaction pathway (II) and 0.5 eV for (III). This trend is indicative of a change in the oxidation state of the Mg center. Furthermore, it is possible to distinguish between different species in solution by peak assignment.

The same blue shift reported for the K‐edge is reproduced at the L_1_‐edge, with 0.6 eV for (II) and 0.5 eV for (III). Studying the reaction at the L_1_‐edge still provides the selectivity of the K‐edge. Additionally, it gives access to the XUV energy range, which may be more readily accessible in experiments. However, the Mg L_1_‐edge may pose an experimental challenge due to the significant carbon and oxygen background in THF.

We used the ΔKohn‐Sham method to estimate the binding energies of Mg 1s. We used three exchange‐correlation functionals and found that they describe the same spectral pattern when progressing from reagents to intermediates of reaction to products. In particular, we observe a blue shift of 0.7 eV for reaction (II) and 0.8 eV for (III). Furthermore, XPS spectra display only a single peak per magnesium atom (or two for the dimer structures), simplifying species identification. From the analysis of the chemical shifts, it became clear that an increase in the binding energy is accompanied by a progressively more positive partial charge on the magnesium atom. This serves as an indicator of the geometric transformations that take place within the first solvated sphere.

In this study, we have focused on time‐independent spectra, as they would apply to an equilibrium in solution. This allows to characterize intermediates of the reaction, which are stationary states on the potential energy surface. However, it does not allow for access to transition states or any other transient structures. The next possible step could be to employ ultrafast X‐ray spectroscopy, which would enable tracking a specific reaction pathway along its reaction coordinate. Such a measurement would require a pump‐probe technique that launches the reaction, for example, with an ultrashort infrared pulse by exciting the carbonyl bond.^[41]^ The X‐ray probe pulse could then probe transient species and reveal new details on the exact reaction mechanism. Furthermore, time‐resolved X‐ray absorption spectroscopy (TR‐XAS) or different static techniques, such as resonant inelastic X‐ray scattering (RIXS), may assist in differentiating the signals originating from the monomeric species from those associated with the dimer reaction pathway, where one of the magnesium centers becomes hexa‐coordinated.

## Computational Details

Geometry optimizations and frequency analysis were performed at the B3LYP/aug‐CC‐PVDZ[^42^, ^43^, ^44^, ^45^, ^46^, ^47^, ^48^] level of theory with a pruned (99,590) grid using Gaussian 16 Rev.C.01 program package.^[49]^ X‐ray absorption (XA) at M K‐ and L_1_‐edges was modeled by linear response density functional theory (LR‐DFT) calculations at the Tamm‐Dancoff approximation (TDA)^[50]^ as implemented in the ORCA 5.0.4 program package.[^51^, ^52^] We computed 25 roots using the relativistic recontracted basis set DKH‐def2‐TZVP[^53^, ^54^] with two different xc functionals: CAM‐B3LYP^[55]^ and *ω*B97X‐D3.^[56]^ In range‐separated functionals, the asymptotic contribution of Hartree‐Fock exchange, $E_{x}^{HF}$ , goes from 65 % in CAM‐B3LYP to 100 % in *ω*B97X‐D3. The effect of the basis set on the calculated roots is shown in Figure S1 of the Supporting Information (SI). The def2‐TZVP is more reliable for the calculation of core‐hole transitions^[57]^ and has been adopted in all LR‐DFT calculations. The SCF calculation was accelerated with the resolution of identity (RI) and chain‐of‐sphere (COSX) approximations with the Coulomb‐fitting auxiliary basis set def2/J^[58]^ and the SCF convergence was set to “tight” (Energy change 1.0e–08 au). Quadrupole contributions were also taken into account.

In addition to the LR‐DFT calculations, we computed 20 roots at the Mg K‐edge at the XMS‐RASPT2/ANO‐RCC‐VTZP[^59^, ^60^, ^61^, ^62^, ^63^, ^64^] level of theory based on a RASSCF^[65]^ wave function with active space RAS(12;1;0;1;13;0) and an imaginary shift of 0.35, as implemented in OpenMolcas v.23.02.^[66]^ In the tag RAS$\left(n;l;m;i;j;k\right)$ , *i*, *j*, and *k* are the number of orbitals in the RAS1, RAS2, and RAS3 subspaces, respectively; *n* is the total number of electrons in the active space, *l* the maximum number of holes allowed in the RAS1, and *m* the maximum number of electrons allowed in RAS3. We used the HEXS keyword^[67]^ to calculate both ground, valence‐ and core‐excited states, and corresponding dipole transition moments, within the same active space. The computation of the two‐electron integrals was sped up using Cholesky decomposition.^[68]^ A further benchmark of the X‐ray absorption spectrum was carried out at the DFT/MRCI(2)[^69^, ^70^, ^71^] with QTP17^[72]^ xc functional, QE8 hamiltonian^[71]^ and def2‐TZVP basis set, using the core‐valence separation^[73]^ (CVS) approximation with the GRACI^[74]^ program. The active space RAS(14;2;2;7;0;21) was employed in the calculations. A Gaussian broadening of 0.5 eV (FWHM) was used for all absorption spectra.

Typically, the absolute excitation energies obtained from DFT calculations have errors of several eVs.[^75^, ^76^] To correct for this, it is common practice to shift the energy axis by computing the difference with respect to a reference. The systematic shift for the XAS spectra at the Mg K‐edge was calculated using the near‐edge X‐ray absorption fine structure (NEXAFS) of the tetra‐coordinated MgCl_2_THF_2_ (M4)^[77]^ as a reference. The absorption edge of M4 is observed at 1309.8 eV,^[77]^ producing an offset of 33.3 eV for *ω*B97X‐D3 and 0.29 eV for XMS‐RASPT2. For the magnesium L_1_‐edge, it was more difficult to find a suitable alignment scheme, as we could not find suitable spectra of similar organomagnesium species at the Mg L_1_‐edge, in the literature. Therefore, we used the atomic energy level (89.4 eV) reported in Ref. [78] as a benchmark and shifted our energy axis by 6.7 eV (*ω*B97X‐D3/def2‐TZVP).

The Mg 1s ionization potentials (IP) were calculated using the Delta Kohn‐Sham method[^79^, ^80^, ^81^] (ΔK‐S) with B3LYP, M06 and M06‐2X[^82^, ^83^] using OpenMolcas. With ΔK‐S, the transition energy is determined by the total energy of the core‐ionized state minus the energy of the ground state. The calculated energies have been convoluted with a Lorentzian band with γ=0.4 eV (half bandwidth at half peak height) to generate the X‐ray photoelectron (XP) spectra. The LoProP approach^[84]^ was used to calculate the localized atomic charges on the metal center.

Similar to the L_1_‐edge case, there are no XPS spectra of similar organomagnesium species in THF reported in the literature, to our knowledge. Therefore, we compared our calculations with the solid phase MgF_2_, which has an experimental Mg 1s binding energy of 1305 eV.^[85]^ The alignment scheme introduces an offset of 29.51 eV (B3LYP), 30.95 eV (M06) and 38.79 eV (M06‐2X).

All calculations were carried out in THF. In the calculation carried out with Gaussian 16, Orca and OpenMolcas, the effect of bulk solvent was represented by the polarizable continuum model (PCM).[^86^, ^87^, ^88^, ^89^] In GRACI, the effect of bulk solvent was described with the COnductor‐like Screening MOdel (COSMO).^[90]^ The solvent molecules actively coordinating the metal center were explicitly considered in the calculations. The exact number of solvent molecule coordinating the Mg atom in each species is displayed in Figure 1. This increased computational effort, but improved the description of the solvation of organomagnesium species. All calculations of spectra took into account the second‐order Douglas‐Kroll Hess (DKH2) correction[^56^, ^91^, ^92^, ^93^, ^94^, ^95^] to include scalar relativistic effects.

All ORCA and Molcas calculations were performed in a reproducible environment using the Nix package manager together with NixOS‐QChem^[96]^ (commit fac7f800 and nixpkgs commit eba64e14).

## Conflict of Interests

The authors declare no conflict of interest.

1

## Supporting information

As a service to our authors and readers, this journal provides supporting information supplied by the authors. Such materials are peer reviewed and may be re‐organized for online delivery, but are not copy‐edited or typeset. Technical support issues arising from supporting information (other than missing files) should be addressed to the authors.

Supporting Information

## Data Availability

Adidditional data that support the findings of this study are available in the supplementary material of this article.
