# Supplementary material for: Distinguishing Organomagnesium Species in the Grignard Addition to Ketones with X‐Ray Spectroscopy
Source: Chemistry. 2024 Nov 7;30(70):e202402099. doi: 10.1002/chem.202402099 (PMC11639641; doi:10.1002/chem.202402099)
Supplement: Supplementary file 1 — Supporting Information [file CHEM-30-e202402099-s001.pdf]

Chemistry–A European Journal

Supporting Information

Distinguishing Organomagnesium Species in the Grignard Addition to Ketones with X-Ray Spectroscopy

Lorenzo Restaino,* Riccardo Mincigrucci, and Markus Kowalewski*

Distinguishing Organomagnesium Species in the Grignard Addition to Ketones with X-ray Spectroscopy

Lorenzo Restaino,[1] Riccardo Mincigrucci,[2] and Markus Kowalewski[1,*]

[1]*Department of Physics, Stockholm University, Albanova University Centre, SE-106 91 Stockholm, Sweden*

[2]*Elettra Sincrotrone Trieste SCpA, Strada Statale 14 - km 163, 5 in AREA Science Park 34149 Basovizza, Trieste Italy*

CONTENTS

S1. Natural Transition Orbitals 2

S2. Comparison Between Methods 6

A. XMS-RASPT2 Active Spaces 8

B. DFT/MRCI(2) Active Spaces 8

S3. Additional X-ray Absorption Spectra 11

A. Mg K-edge 11

B. Mg L_1-edge 12

S4. Solvent Effect on the Magnesium Cation 17

S5. Additional Correlation Plots 18

S6. Cartesian Coordinates of Optimised Minima Structures 19

* e-mail: markus.kowalewski@fysik.su.se

S1. NATURAL TRANSITION ORBITALS

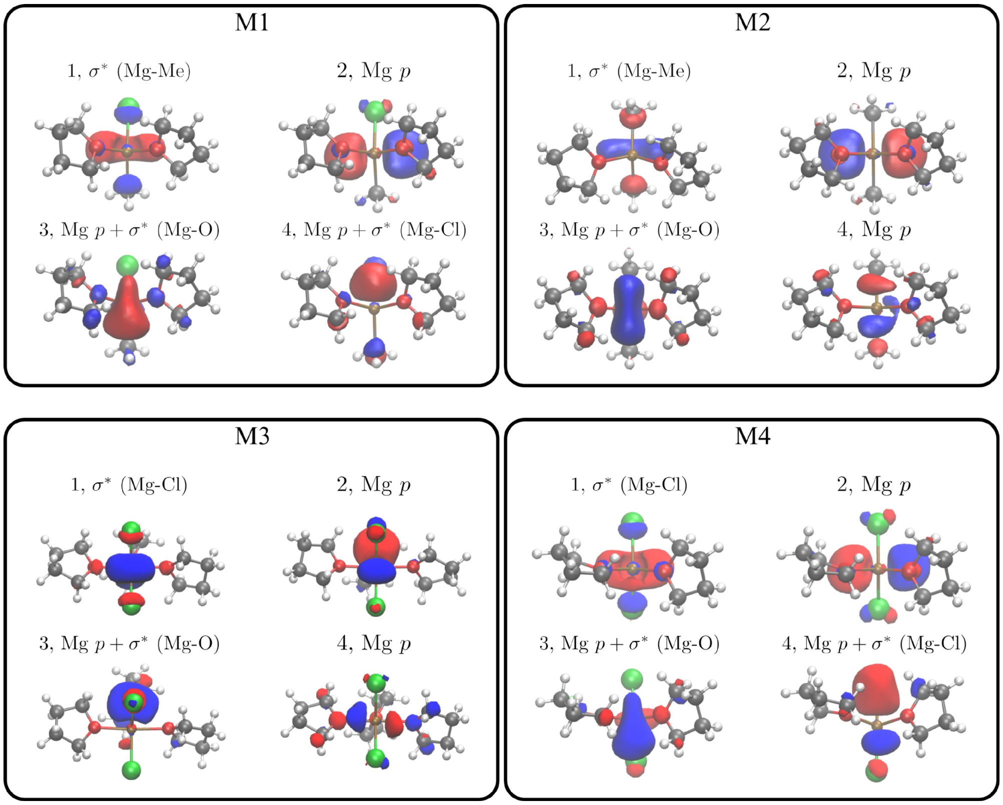

Figure S1. Natural transition orbitals (NTOs) for reaction pathway I plus reference molecule (M4), calculated at the TDA/ωB97X-D3/DKH-def2-TZVP level of theory. The corresponding peak positions are reported in Table 1 of the article.

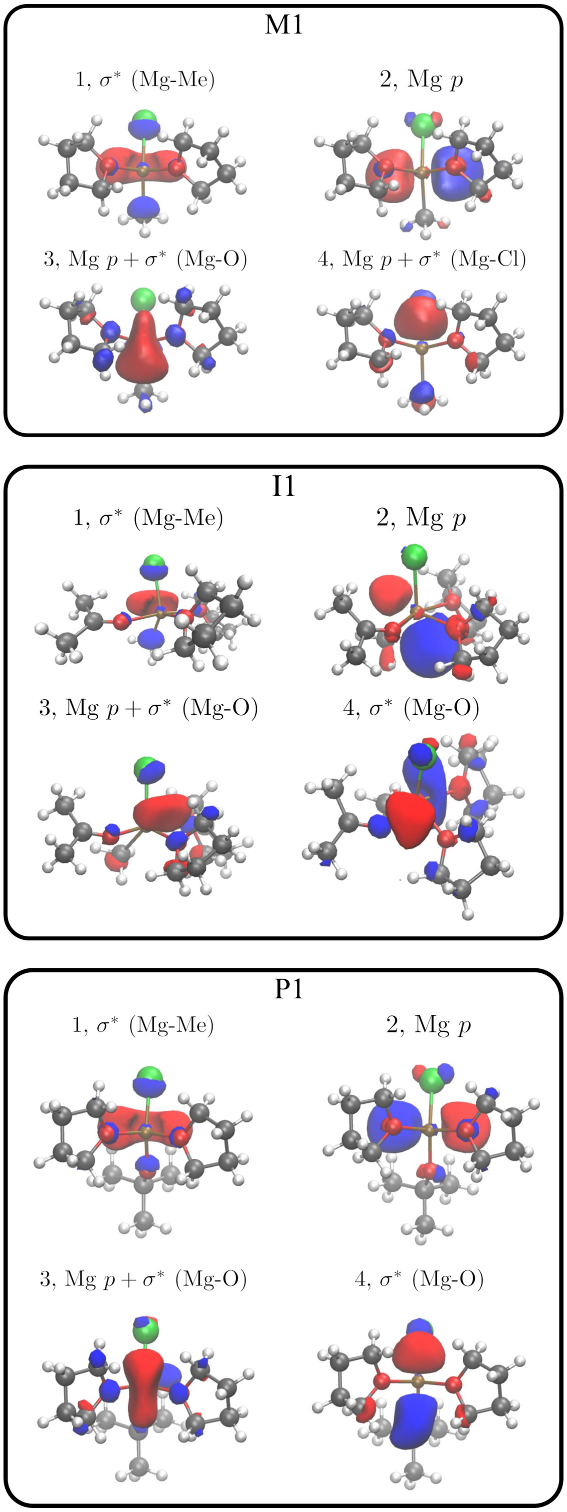

Figure S2. Natural transition orbitals (NTOs) for reaction pathway II, calculated at the TDA/ωB97X-D3/DKH-def2-TZVP level of theory. The corresponding peak positions are reported in Table 1 of the article.

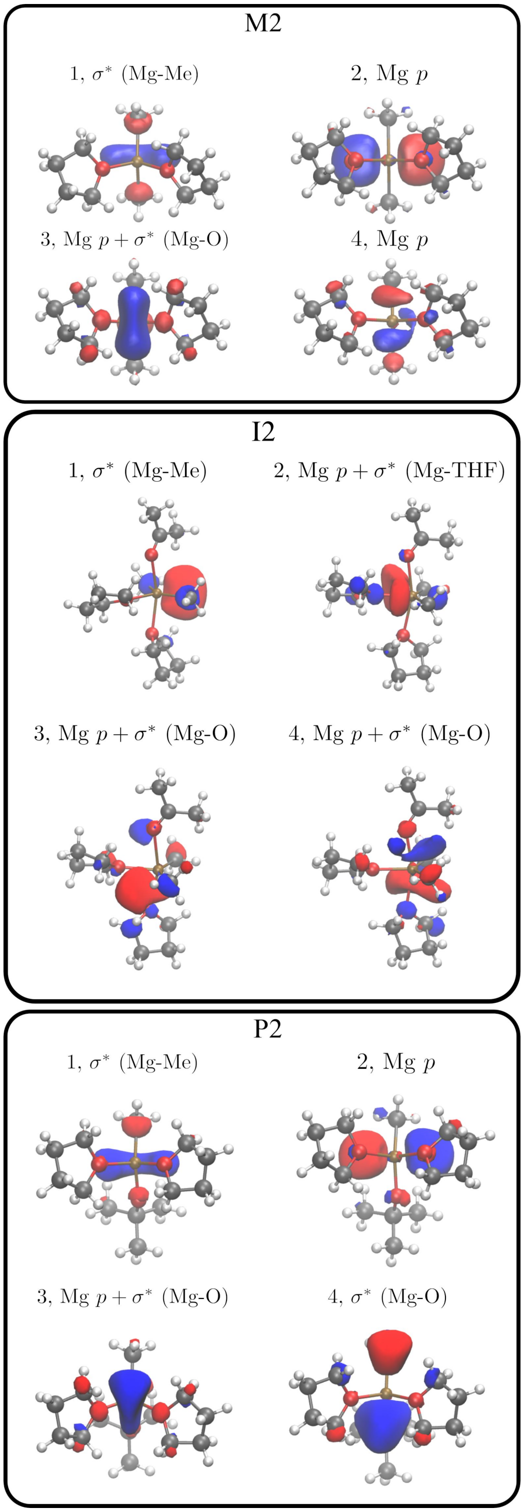

Figure S3. Natural transition orbitals (NTOs) for reaction pathway III, calculated at the TDA/ωB97X-D3/DKH-def2-TZVP level of theory. The corresponding peak positions are reported in Table 1 of the article.

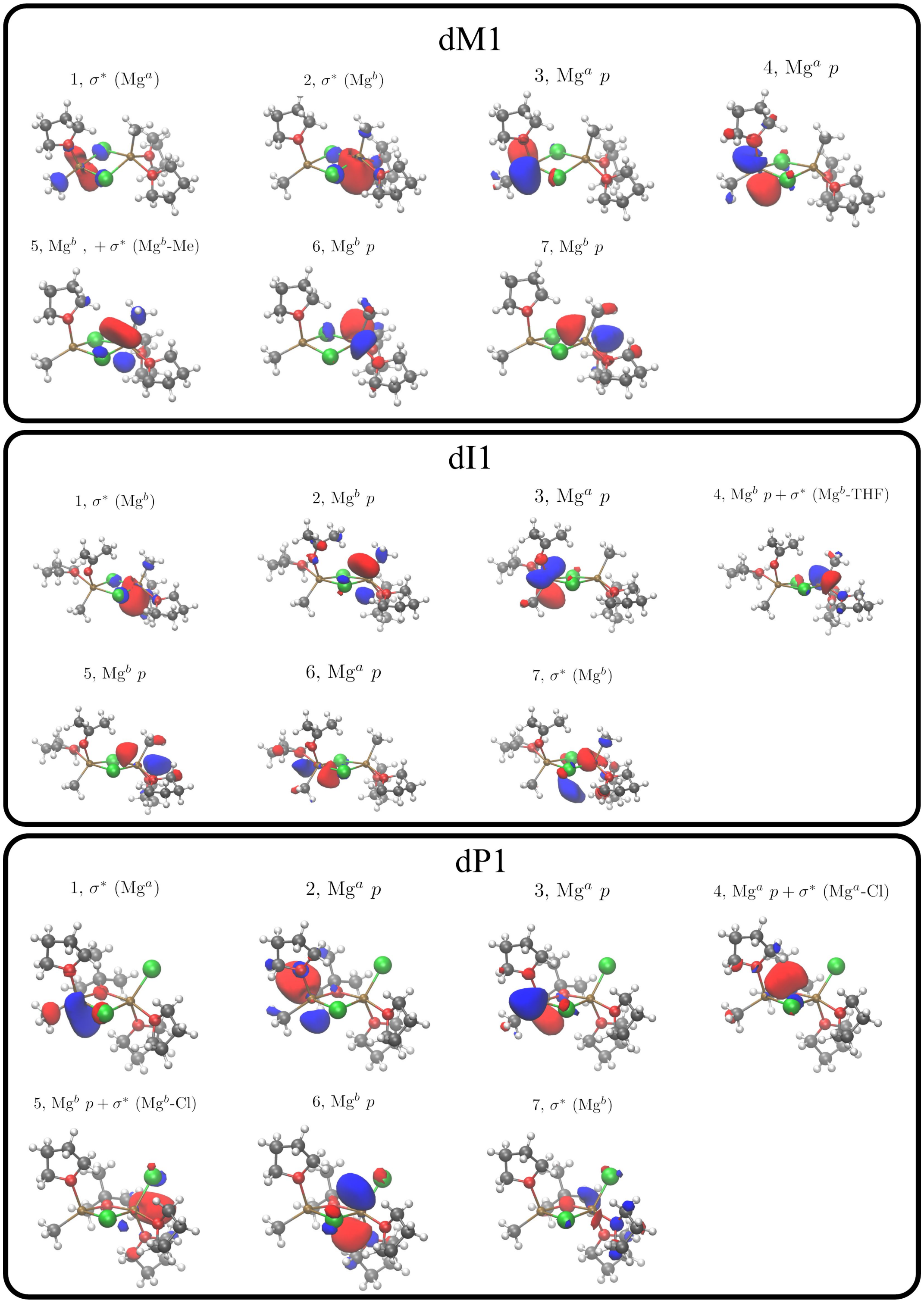

Figure S4. Natural transition orbitals (NTOs) for reaction pathway IV, calculated at the TDA/ωB97X-D3/DKH-def2-TZVP level of theory. The corresponding peak positions are reported in Table 1 of the article.

S2. COMPARISON BETWEEN METHODS

The effect of the basis set on the X-ray absorption spectrum of M4 ($MgCl_2THF_2$) calculated at the TDA/ωB97X-D3 level of theory is displayed in fig. S5. Due to the alignment scheme discussed in the main text, we applied a 29.7 eV and 33.3 eV shift of the energy axis for aug-CC-PVTZ and def2-TZVP, respectively. Despite both basis sets replicating the pre-edge, first strong peak, and right shoulder, differences are noticeable in the calculated spectra for photon energies above 1311 eV.

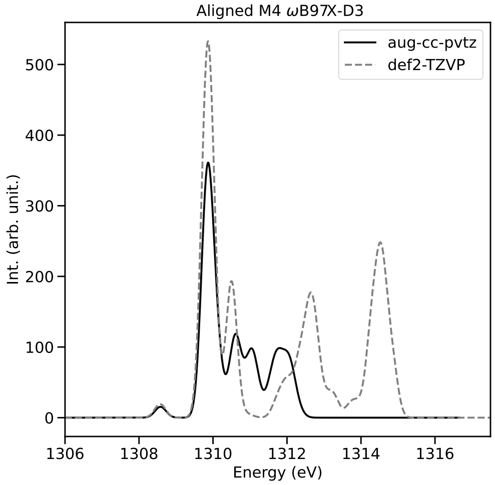

Figure S5. Comparison between XAS of M4 calculated with two basis set: aug-CC-PVTZ (solid black line) and def2-TZVP (dashed gray line). Spectra shifted according to the alignment scheme.

A comparison between the LR-DFT and XMS-RASPT2 calculated absorption spectrum of M1 is displayed in fig. S6. The offset in the energy axis introduced by the alignment scheme was 0.29 eV. There is good agreement between the two methods. The XMS-CASPT2 spectrum is blue-shifted by approximately 2 eV with respect to LR-DFT.

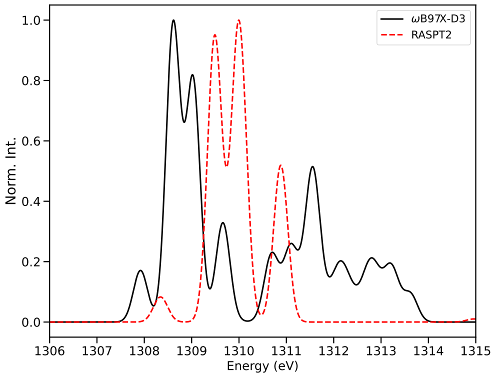

Figure S6. Comparison between X-ray absorption spectra of M1 ($CH_3MgClTHF_2$) calculated with TDA/ωB97X-D3/def2-TZVP (solid, black) and XMS-RASPT2/ANO-RCC-VTZP (dashed, red). The signals are normalised with respect to their maximum intensity to compare different methods.

A. XMS-RASPT2 Active Spaces

In this section we display the active space used in the XMS-RASPT2 calculations of M4 and M1.

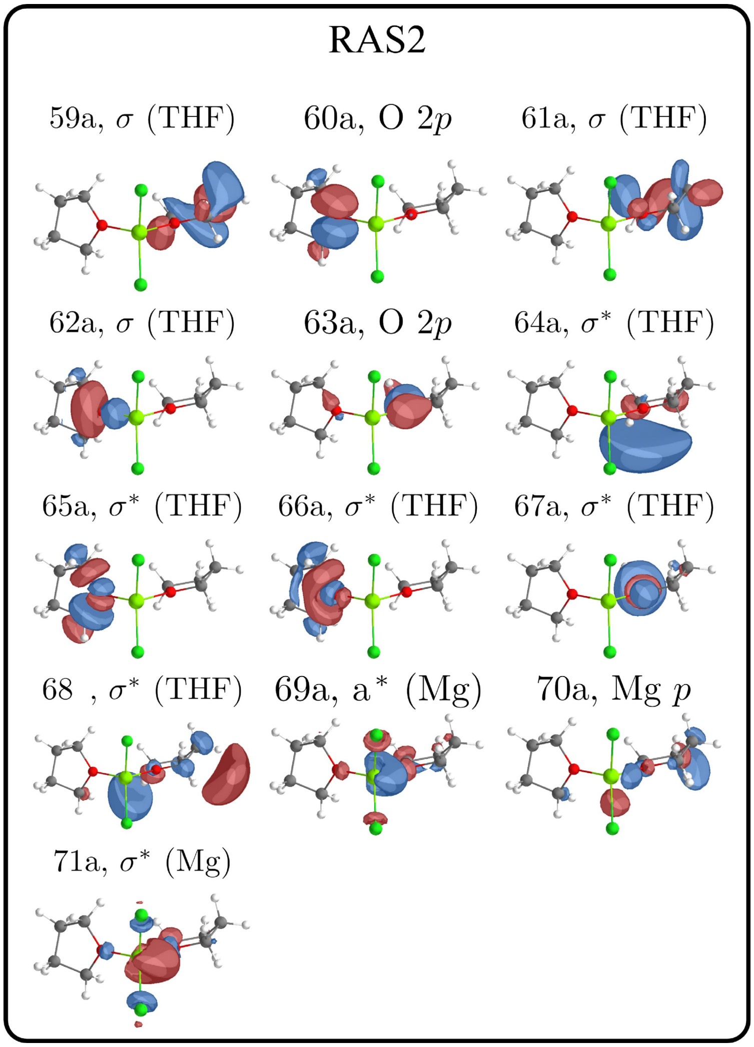

Figure S7. RAS2 active space used in the XMS-RASPT2/ANO-RCC-VTZP calculation of M4 ($(Cl)_2MgTHF_2$) in C_1 symmetry. The RAS1 contains only the Mg 1s.

B. DFT/MRCI(2) Active Spaces

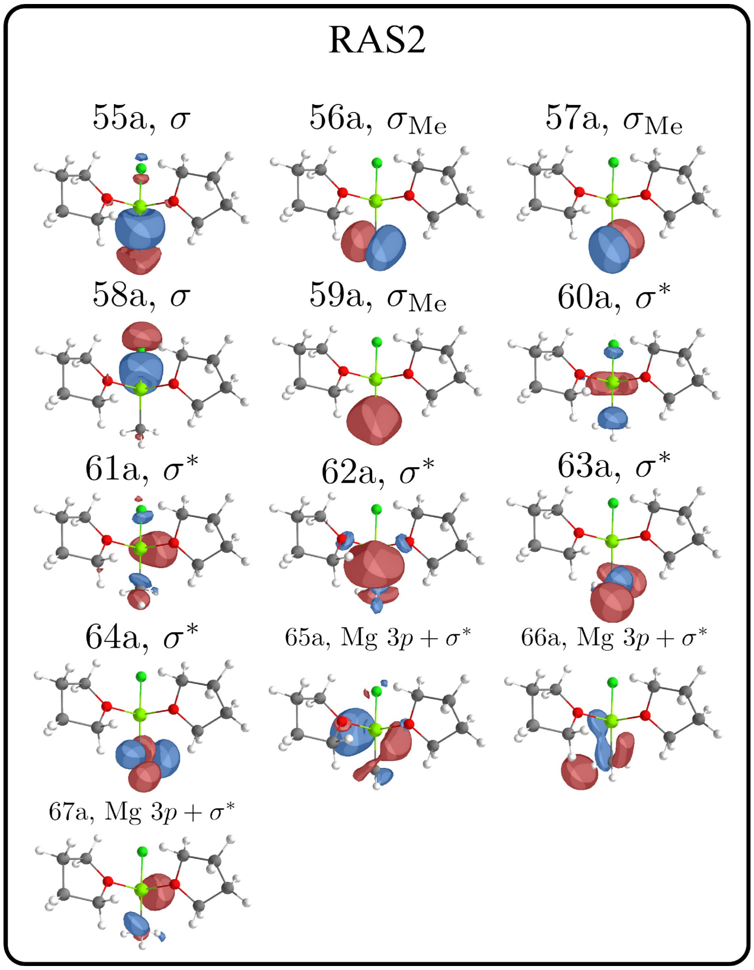

Figure S8. RAS2 active space used in the XMS-RASPT2/ANO-RCC-VTZP calculation of M1 ($CH_3MgClTHF_2$) in C_1 symmetry. The RAS1 contains only the Mg 1s.

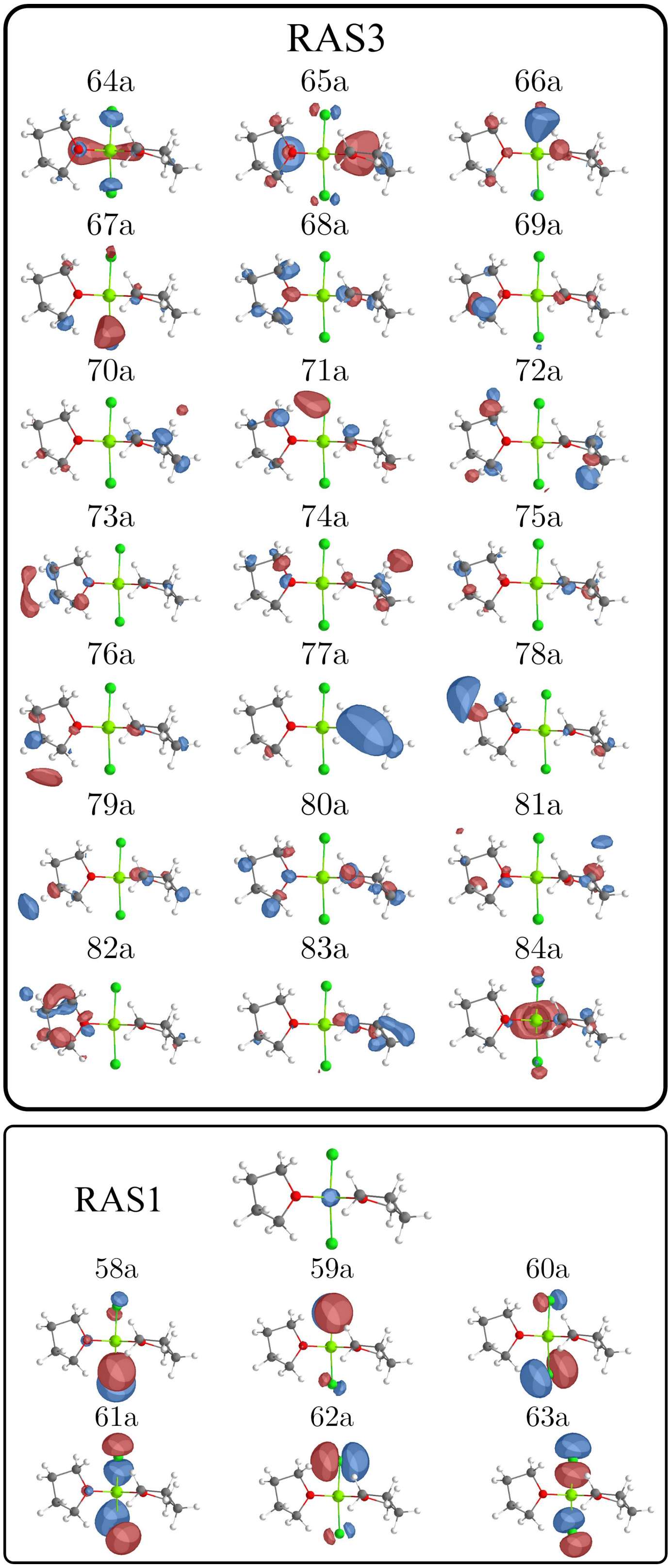

Figure S9. RAS1 and RAS3 used in the DFT/MRCI(2) calculation of M4 ($(Cl)_2MgTHF_2$) in C_1 symmetry. The RAS2 is empty.

S3. ADDITIONAL X-RAY ABSORPTION SPECTRA

In this section we show the X-ray absorption spectra of the isolated molecules in tetrahydrofuran at different stages of the reaction mechanism.

A. Mg K-edge

Figure S10 shows the evolution of the absorption spectra of M1 ($CH_3MgClTHF_2$) following the reaction pathway (II). Figure S11 shows the evolution of the absorption spectra of M2 ($(CH_3)_2MgTHF_2$) following the reaction pathway (III). Figure S12 shows the evolution of the absorption spectra of the binuclear structure dM1 following the reaction pathway (IV).

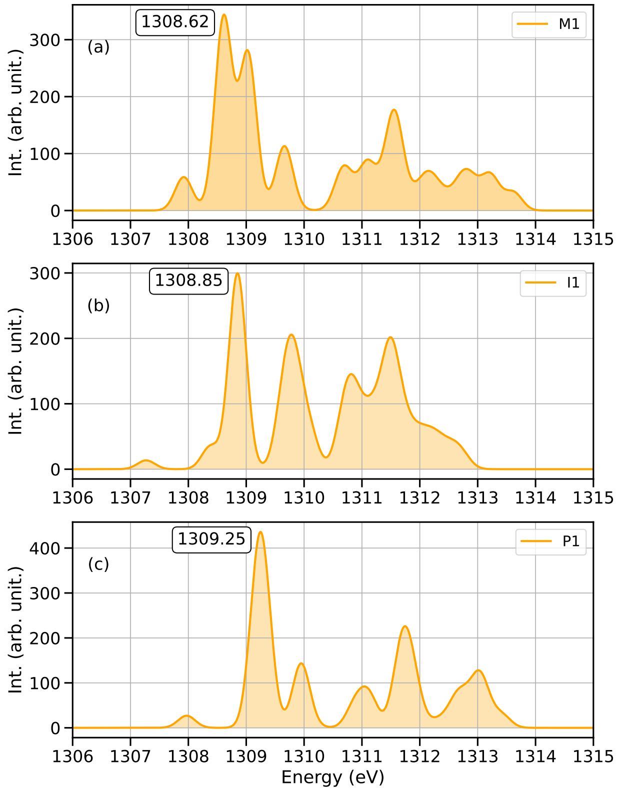

Figure S10. Calculated X-ray absorption spectra at Mg K-edge for different stages of reaction (II): reactant M1 (a), intermediate of reaction I1 (b), and product P1 (c) in THF.

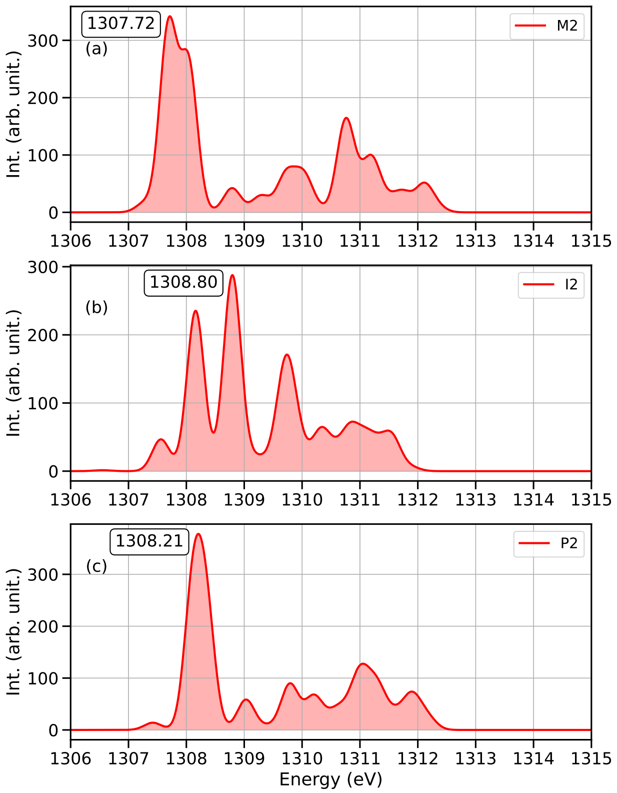

Figure S11. Calculated X-ray absorption spectra at Mg K-edge for different stages of reaction (III): reactant M2 (a), intermediate of reaction I2 (b), and product P2 (c) in THF.

B. Mg L_1-edge

Figure S13 shows the evolution of the absorption spectra of M1 ($CH_3MgClTHF_2$) following the reaction pathway (II). Figure S14 shows the evolution of the absorption spectra of M2 ($(CH_3)_2MgTHF_2$) following the reaction pathway (III). Figure S16 shows the evolution of the absorption spectra of the binuclear structure dM1 following the reaction pathway (IV).

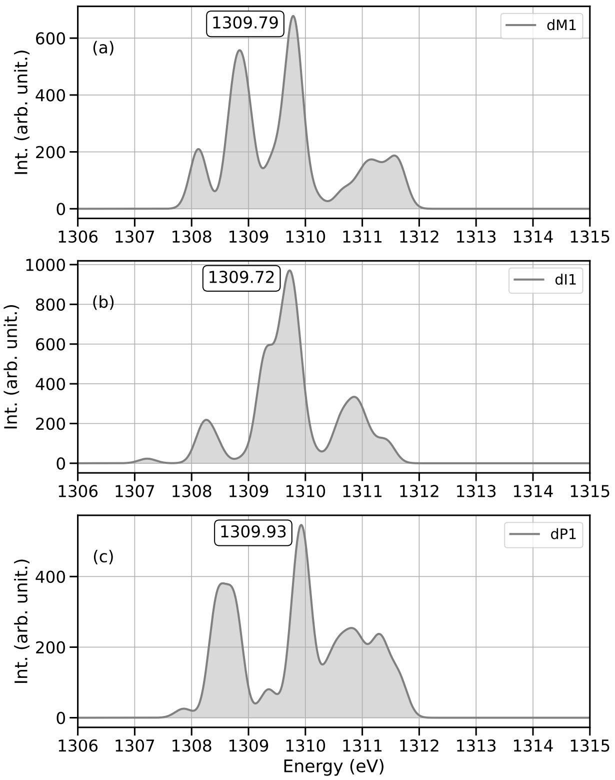

Figure S12. Calculated X-ray absorption spectra at Mg K-edge for different stages of reaction (IV): reactant dM1 (a), intermediate of reaction dI1 (b), and product dP1 (c) in THF.

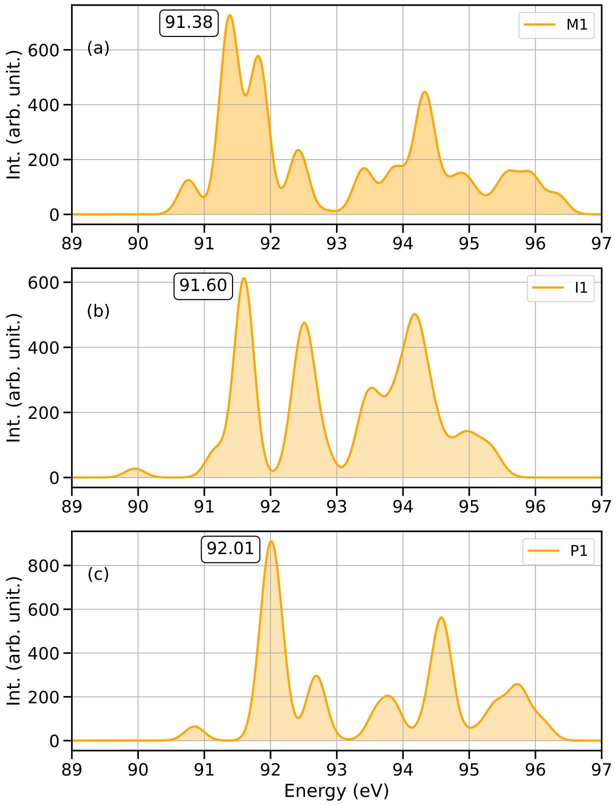

Figure S13. Calculated X-ray absorption spectra at Mg L_1-edge for different stages of reaction (II): reactant M1 (a), intermediate of reaction I1 (b), and product P1 (c) in THF.

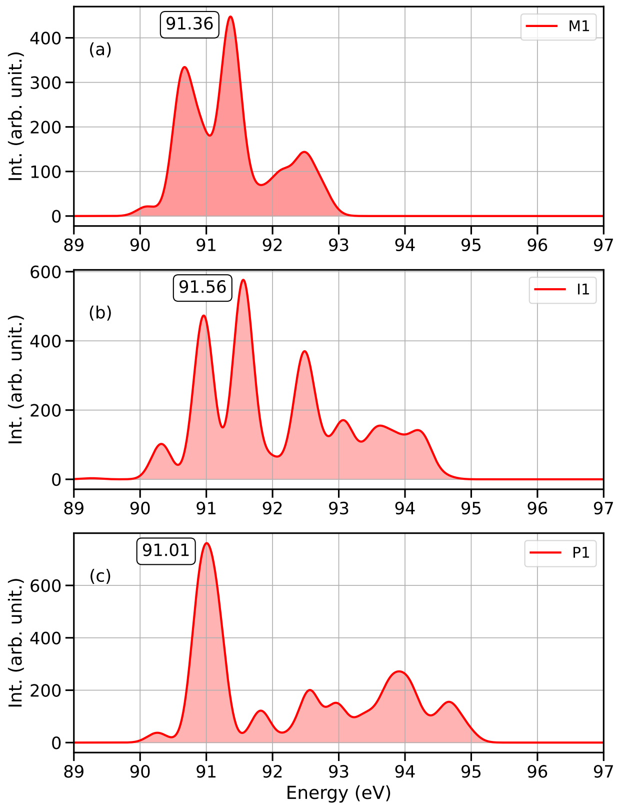

Figure S14. Calculated X-ray absorption spectra at Mg L_1-edge for different stages of reaction (III): reactant M2 (a), intermediate of reaction I2 (b), and product P2 (c) in THF.

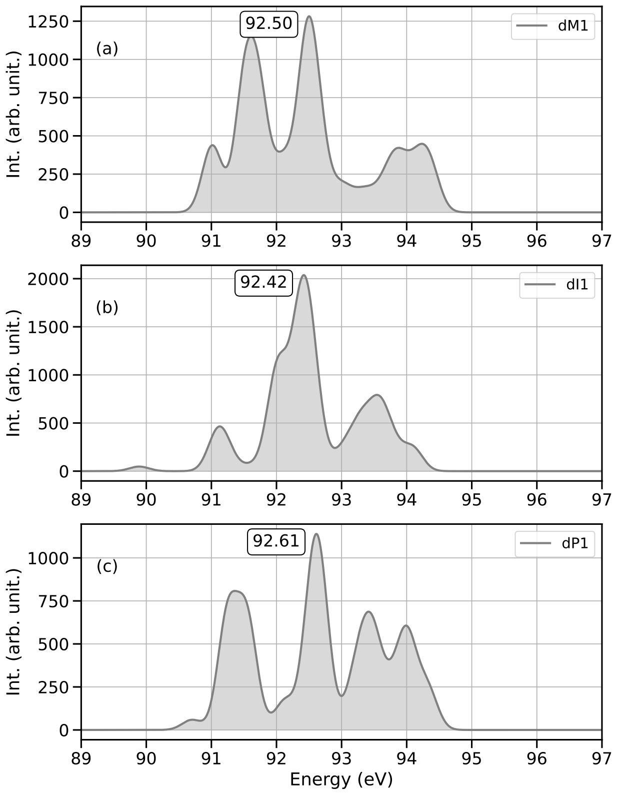

Figure S15. Calculated X-ray absorption spectra at Mg L_1-edge for different stages of reaction (IV): reactant dM1 (a), intermediate of reaction dI1 (b), and product dP1 (c) in THF.

S4. SOLVENT EFFECT ON THE MAGNESIUM CATION

Table S1. Alignment scheme for the MgF_2 1s binding energies calculated at the ΔK-S B3LYP level of theory with and without polarizable continuum model (PCM). The stabilizing effect due to the PCM amounts to 4.35 eV.

	NO PCM	With PCM
GS Energy (H)	-400.51	-400.57
ION Energy (H)	-351.47	-351.68
BE (H)	49.04	48.88
BE (eV)	1334.52	1330.16
Exp. (eV)	1305.00	1305.00
Δ	-29.52	-25.16

S5. ADDITIONAL CORRELATION PLOTS

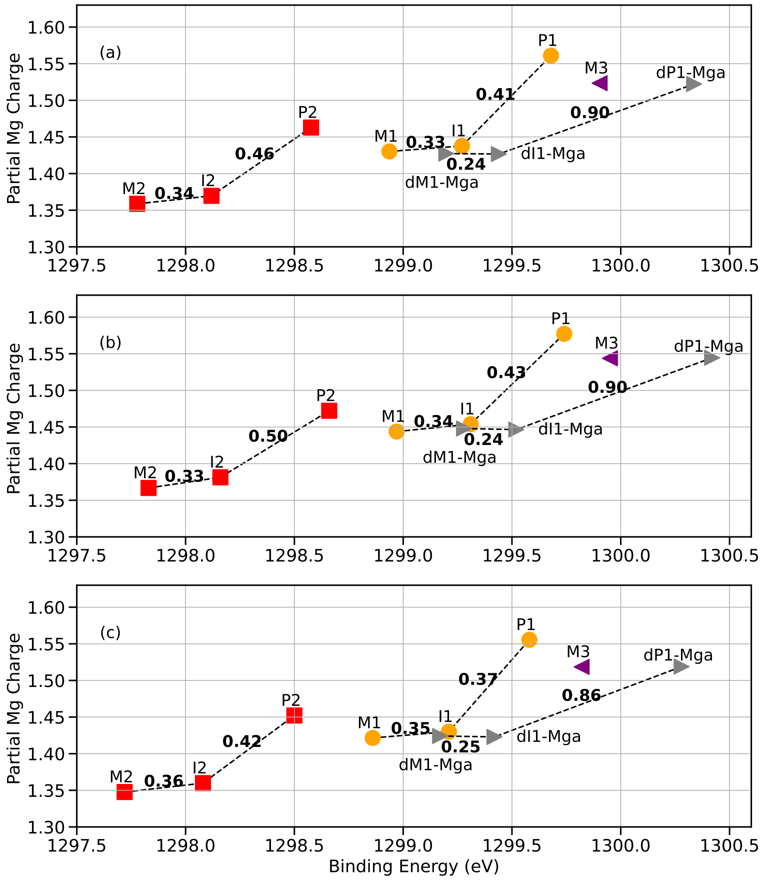

Figure S16. Correlation between the ΔK-S binding energies (eV) of Mg 1s electron and local partial charge on Mg atom for different species in solution. (a) B3LYP as reported in the main text; (b) M06; (c) M06-2X. Dashed lines indicate ΔBEs reported in bold.

S6. CARTESIAN COORDINATES OF OPTIMISED MINIMA STRUCTURES

```
   32
M1 - B3LYP/aug-CC-PVTZ
 C      2.065780    -2.352471    -0.102123
 O      1.307365    -1.339691    -0.834720
 C      1.396519    -1.581703    -2.274024
 C      2.480490    -2.643962    -2.436112
 C      2.367071    -3.441455    -1.126717
Mg     -0.010086    -0.002739     0.085367
 C      0.356211    -0.038211     2.195101
Cl     -2.014584    -0.271560    -1.173051
 H      1.434498     0.021767     2.435303
 H     -0.124992     0.800436     2.731014
 H     -0.013183    -0.961526     2.678562
 H      3.470703    -2.175654    -2.515643
 H      2.315700    -3.259299    -3.327926
 H      3.282286    -3.991527    -0.879616
 H      1.535204    -4.155980    -1.182143
 H      1.452252    -2.685447     0.741027
 H      2.981093    -1.879765     0.279017
 H      1.631353    -0.626574    -2.756745
 H      0.414201    -1.930048    -2.616908
 O      0.832789     1.685027    -0.811128
 C      2.176730     2.141241    -0.462893
 C      0.178149     2.655056    -1.689119
 C      2.239195     3.594372    -0.924240
 H      2.304003     2.008653     0.616937
 H      2.897106     1.506603    -0.997013
 C      1.289928     3.600951    -2.133250
 H     -0.294566     2.097753    -2.503161
 H     -0.595259     3.171743    -1.105548
 H      3.261207     3.897209    -1.179085
 H      1.864575     4.262326    -0.137380
 H      1.797914     3.206561    -3.023508
 H      0.903871     4.599372    -2.368468
```

42

I1 – B3LYP/aug–CC–PVTZ

C	1.482491	1.106454	2.267641
O	1.685099	0.084969	1.250300
C	2.717426	−0.847113	1.682850
C	2.927329	−0.568555	3.169245
C	2.626486	0.935078	3.266506
Mg	−0.155582	−0.619158	0.183107
O	−2.077794	−0.682440	−0.909319
C	−2.911074	−1.511159	−1.272758
C	−1.111901	−1.369188	1.975704
Cl	1.133383	−1.787795	−1.490148
H	−1.805395	−0.635186	2.430282
H	−1.724145	−2.266392	1.764187
H	−0.406787	−1.657367	2.777612
H	3.941581	−0.826104	3.496577
H	2.210594	−1.143365	3.770562
H	3.500974	1.522035	2.954315
H	2.339493	1.253479	4.275588
H	0.502006	0.932678	2.732107
H	1.478427	2.082717	1.769318
H	3.624465	−0.647034	1.094918
H	2.370229	−1.862172	1.464918
O	−0.132781	1.390191	−0.511915
C	0.933158	1.919911	−1.358954
C	−1.205105	2.368547	−0.377669
C	0.621332	3.405748	−1.513786
H	1.885266	1.707099	−0.863104
H	0.899285	1.386931	−2.317770
C	−0.913156	3.433205	−1.432254
H	−2.153039	1.842035	−0.522609
H	−1.163675	2.780505	0.640673
H	1.008851	3.806313	−2.457642
H	1.059891	3.980813	−0.687086
H	−1.354002	3.147692	−2.396772
H	−1.311583	4.413169	−1.145131
C	−4.333186	−1.092845	−1.536982
H	−4.682827	−1.493347	−2.498283
H	−4.977827	−1.528724	−0.758111
H	−4.427162	−0.002923	−1.518888
C	−2.564460	−2.962054	−1.458078
H	−3.271298	−3.589995	−0.897673
H	−2.683930	−3.222095	−2.520650
H	−1.535886	−3.162220	−1.148009

```
    42
P1 - B3LYP/aug-CC-PVTZ
 C      -0.048937      -0.074973      -0.095862
 O       0.263963      -0.025944       1.333661
 C       1.704008       0.121588       1.534161
 C       2.326412      -0.186951       0.177055
 C       1.249205       0.310376      -0.801088
Mg      -1.123471      -0.274418       2.849936
 O      -1.239756       1.690490       3.487082
 C      -0.546731       2.261653       4.643078
 C      -1.135145       3.661657       4.820834
 C      -2.537053       3.528882       4.203212
 C      -2.283928       2.602275       3.021568
 O      -2.799627      -0.528584       2.035020
Cl       0.051120      -1.372883       4.532543
 H      -0.542716       4.402165       4.266138
 H      -1.157178       3.959151       5.875386
 H      -2.956950       4.491555       3.888408
 H      -3.231530       3.061360       4.913981
 H      -3.132299       1.981792       2.720984
 H      -1.896483       3.150279       2.150588
 H       1.996539      -0.566845       2.333627
 H       1.898443       1.155217       1.850666
 H       2.481067      -1.268161       0.063519
 H       3.290314       0.317757       0.046395
 H       1.324540      -0.150029      -1.792417
 H       1.313167       1.400228      -0.919009
 H      -0.879669       0.612358      -0.279275
 H      -0.364276      -1.098492      -0.334707
 H      -0.744497       1.609800       5.501903
 H       0.527851       2.259267       4.428250
 C      -3.808697      -1.467424       1.777375
 C      -4.571432      -1.823585       3.071766
 H      -5.377345      -2.548522       2.876329
 H      -3.885469      -2.266177       3.807817
 H      -5.019190      -0.922860       3.516284
 C      -3.224348      -2.774581       1.201262
 H      -4.018495      -3.505985       0.983165
 H      -2.676359      -2.574258       0.269736
 H      -2.529428      -3.232913       1.920866
 C      -4.811247      -0.887146       0.756361
 H      -4.300838      -0.654132      -0.189299
 H      -5.624707      -1.596649       0.543394
 H      -5.255420       0.041889       1.143546
```

```
35
M2 - B3LYP/aug-CC-PVTZ
Mg     0.113287     1.528548    -0.047846
 C     0.169893     2.373897    -2.042166
 H     0.211938     1.598656    -2.837919
 H    -0.714453     2.992655    -2.270365
 H     1.044931     3.028153    -2.212602
 C     0.071706     2.265109     1.988890
 O     1.668144     0.064916    -0.069543
 C     2.082590    -0.696057     1.103546
 C     3.163762    -1.653156     0.605443
 H     2.713259    -2.585562     0.239195
 H     3.882979    -1.903015     1.394016
 C     3.792123    -0.868606    -0.557549
 H     4.313119    -1.510822    -1.277093
 H     4.503668    -0.122919    -0.178133
 C     2.580698    -0.180037    -1.179803
 H     2.801883     0.783968    -1.648752
 H     2.069958    -0.826536    -1.907325
 H     2.467654     0.013237     1.850614
 H     1.195690    -1.199896     1.508912
 H     0.006772     1.521284     2.681906
 H     1.002930     2.871070     2.259825
 H    -0.834892     2.978893     2.198622
 O    -1.484031     0.108401    -0.098148
 C    -2.447169    -0.114060     0.973365
 C    -1.922552    -0.559441    -1.318745
 C    -3.694197    -0.659113     0.283089
 H    -2.015645    -0.842563     1.677229
 H    -2.597366     0.840306     1.490851
 C    -3.100243    -1.438383    -0.901361
 H    -2.215786     0.213481    -2.041058
 H    -1.070584    -1.120631    -1.719191
 H    -4.324468     0.165507    -0.076272
 H    -4.292794    -1.286949     0.953276
 H    -3.812652    -1.582841    -1.721826
 H    -2.746718    -2.424772    -0.571962
```

45
I2 – B3LYP/aug–CC–PVTZ

C	−0.287738	5.855134	0.018098
C	−0.431991	4.609828	−0.854547
O	0.765124	3.823055	−0.618456
C	1.764716	4.632860	0.056539
C	1.234303	6.067596	0.027611
Mg	0.525219	1.587590	−0.042432
O	0.999768	−0.669143	0.354641
C	0.322477	−1.656750	0.626111
C	0.976186	−2.983952	0.912882
C	0.406475	1.984730	2.101563
O	2.567377	1.549128	−0.686861
C	3.701942	1.159498	0.137478
C	4.807595	0.773005	−0.844879
C	4.484870	1.641376	−2.071869
C	2.958630	1.629331	−2.084826
C	−1.181131	−1.593507	0.699587
C	−0.837426	1.202871	−1.706742
H	−0.698278	0.205262	−2.166436
H	−1.906156	1.250642	−1.416749
H	−0.720810	1.937507	−2.526359
H	4.736593	−0.292394	−1.101764
H	5.805754	0.963488	−0.433679
H	4.906724	1.244596	−3.002614
H	4.859127	2.664165	−1.929069
H	2.503902	2.533339	−2.503635
H	2.560431	0.748601	−2.608527
H	3.983206	2.024321	0.754367
H	3.378658	0.338847	0.785598
H	1.410755	2.063379	2.564177
H	−0.115568	2.925996	2.362165
H	−0.114289	1.182977	2.660240
H	1.871893	4.254557	1.082184
H	2.716631	4.508305	−0.473769
H	−0.474982	4.865659	−1.924029
H	−1.298677	3.988357	−0.604935
H	1.554257	6.578713	−0.890904
H	1.583646	6.652530	0.886800
H	−0.840029	6.711015	−0.388122
H	−0.654166	5.654968	1.033965
H	−1.632932	−2.508238	0.294854
H	−1.469362	−1.533197	1.760802
H	−1.555091	−0.709455	0.175439
H	0.565792	−3.421185	1.834007
H	0.735300	−3.682320	0.096733
H	2.061915	−2.873825	0.992448

45

P2 – B3LYP/aug–CC–PVTZ

C	−0.117136	0.211972	−0.099048
O	0.255106	−0.010434	1.295606
C	1.703590	−0.119513	1.421143
C	2.209126	−0.343799	−0.000065
C	1.198669	0.457801	−0.836151
Mg	−1.070065	−0.320527	2.914289
O	−1.165699	1.711661	3.498808
C	−0.861318	2.189000	4.844268
C	−1.388470	3.622823	4.897146
C	−2.551137	3.593342	3.891560
C	−2.014164	2.673156	2.800797
O	−2.767222	−0.497683	2.083889
C	0.048032	−1.363274	4.434317
H	−0.615228	4.329447	4.566787
H	−1.702575	3.903252	5.909181
H	−2.805895	4.586338	3.503006
H	−3.448593	3.158280	4.351393
H	−2.777547	2.096232	2.269992
H	−1.388066	3.218693	2.079809
H	1.914298	−0.942043	2.111811
H	2.084406	0.819750	1.847277
H	2.164461	−1.410347	−0.258325
H	3.241599	0.001679	−0.127089
H	1.147820	0.128465	−1.880358
H	1.453936	1.525925	−0.822103
H	−0.809874	1.059414	−0.132592
H	−0.633215	−0.688198	−0.456144
H	−1.373296	1.533719	5.561141
H	0.221095	2.109297	4.993938
C	−3.750310	−1.466730	1.864334
C	−4.454113	−1.823064	3.193405
H	−5.251039	−2.569421	3.053322
H	−3.725063	−2.228732	3.909646
H	−4.900305	−0.920140	3.634070
H	0.957024	−0.812701	4.743883
H	−0.534467	−1.530935	5.359967
H	0.394998	−2.363205	4.111089
C	−3.126830	−2.748364	1.265650
H	−3.883251	−3.523106	1.067858
H	−2.620164	−2.512910	0.318757
H	−2.382748	−3.166818	1.958937
C	−4.796304	−0.908005	0.875498
H	−4.311334	−0.648732	−0.076655
H	−5.599830	−1.631206	0.669494
H	−5.249962	0.005331	1.286513

42
M3 – B3LYP/aug–CC–PVTZ

Mg	−1.023652	−1.014041	−0.301754
O	−3.081124	−0.742431	0.280054
C	−4.133964	−1.630021	−0.200587
C	−3.671483	0.509001	0.761429
C	−5.416868	−1.099791	0.427291
H	−4.154763	−1.576908	−1.296593
H	−3.872557	−2.647916	0.105653
C	−5.163256	0.415144	0.440702
H	−3.477412	0.569156	1.839309
H	−3.164334	1.340105	0.263192
H	−5.532573	−1.483172	1.450360
H	−6.302942	−1.383036	−0.152762
H	−5.771035	0.946749	1.182085
H	−5.371395	0.844120	−0.548450
Cl	−1.560392	−1.163882	−2.622608
Cl	−0.292465	0.835851	1.040177
O	−1.207062	−2.923162	0.563462
C	−1.005860	−4.175783	−0.163088
C	−1.506822	−3.191967	1.967992
C	−1.508421	−5.265321	0.779154
H	−1.558473	−4.103390	−1.105227
H	0.065425	−4.275179	−0.379071
C	−1.203454	−4.674503	2.165286
H	−0.887007	−2.524816	2.576231
H	−2.565552	−2.958086	2.140557
H	−1.003662	−6.221419	0.600666
H	−2.588822	−5.415001	0.652947
H	−0.145436	−4.818877	2.420309
H	−1.814886	−5.111986	2.962485
O	1.028040	−1.622839	−0.635978
C	1.911122	−1.913721	0.491142
C	1.820212	−1.258931	−1.806250
C	3.322378	−1.568845	0.015816
H	1.578890	−1.311218	1.342645
H	1.806225	−2.981340	0.727998
C	3.224806	−1.767617	−1.504528
H	1.343965	−1.718215	−2.676359
H	1.797306	−0.165460	−1.915894
H	4.078860	−2.207115	0.487213
H	3.557755	−0.522087	0.248684
H	3.311788	−2.831851	−1.763431
H	3.989081	−1.211758	−2.060137

```
      29
M4 -  B3LYP/aug-CC-PVTZ
Mg    -1.863339     0.305306    -0.146708
 Cl   -3.944876     0.311030    -1.211413
 Cl    0.220741     0.685956    -1.161727
  O   -1.880963     1.666121     1.384651
  C   -1.927672     1.377225     2.819066
  C   -1.622596     3.091614     1.160704
  C   -1.893874     2.741164     3.505434
  H   -2.841243     0.806457     3.016993
  H   -1.049271     0.767418     3.065619
  C   -1.120421     3.611333     2.501416
  H   -0.895193     3.165223     0.345961
  H   -2.568873     3.561386     0.861056
  H   -1.408759     2.687293     4.486623
  H   -2.912423     3.127001     3.642074
  H   -0.039239     3.445291     2.595987
  H   -1.320769     4.681796     2.623153
  O   -1.687658    -1.424223     0.923977
  C   -0.418128    -2.120486     1.167761
  C   -2.814810    -2.259122     1.356269
  C   -0.779865    -3.284995     2.081746
  H   -0.027656    -2.456103     0.199193
  H    0.274735    -1.397308     1.608866
  C   -2.209710    -3.630566     1.635748
  H   -3.238155    -1.802036     2.260320
  H   -3.560837    -2.247772     0.555968
  H   -0.769126    -2.968189     3.133067
  H   -0.082027    -4.121664     1.964149
  H   -2.776327    -4.174134     2.400131
  H   -2.191642    -4.235525     0.719888
```

51
dM1 − B3LYP/aug−CC−PVTZ

C	−2.406540	2.436486	1.288004
O	−2.340520	1.329943	0.334625
C	−3.068568	1.670165	−0.880497
C	−3.940706	2.862727	−0.502043
C	−3.067516	3.589224	0.533586
Mg	−0.787712	−0.138538	0.439554
O	−2.389397	−1.510794	−0.031656
C	−2.642312	−2.397416	−1.162822
C	−3.654098	−3.419898	−0.654043
C	−4.464903	−2.603715	0.364096
C	−3.395497	−1.724268	1.006940
Cl	0.705787	−1.446258	−1.164704
Mg	2.336348	0.395041	−1.404468
C	3.403071	0.752193	−3.221230
O	3.669659	−0.062796	0.115231
C	5.051003	−0.465180	−0.149912
C	5.737539	−0.451817	1.212528
C	4.589459	−0.812974	2.168707
C	3.401526	−0.079963	1.554013
Cl	0.790269	1.894613	−0.265933
C	−0.404246	−0.482157	2.524288
H	4.017097	−0.116966	−3.522676
H	4.098355	1.608475	−3.138864
H	2.739897	0.973427	−4.076950
H	5.031006	−1.470427	−0.592029
H	5.471291	0.241843	−0.872433
H	6.124464	0.551007	1.437439
H	6.570636	−1.162789	1.254589
H	4.774746	−0.492436	3.200289
H	4.414447	−1.897080	2.167384
H	3.336412	0.961592	1.894240
H	2.436861	−0.572674	1.709066
H	−0.009519	−1.495048	2.728105
H	0.329510	0.226464	2.949130
H	−1.318865	−0.382781	3.138129
H	−3.050593	−1.793655	−1.986025
H	−1.685067	−2.826480	−1.469876
H	−3.138828	−4.252526	−0.156453
H	−4.267495	−3.826960	−1.466185
H	−4.978383	−3.226967	1.105368
H	−5.215081	−1.986661	−0.148618
H	−2.904975	−2.220456	1.854120
H	−3.757954	−0.741195	1.324859
H	−2.339614	1.929863	−1.661070
H	−3.631290	0.781779	−1.186295
H	−4.880182	2.522013	−0.046367
H	−4.182113	3.484515	−1.371786
H	−3.645825	4.235983	1.203360
H	−2.307936	4.201144	0.029988
H	−3.007214	2.098946	2.142924
H	−1.387584	2.656714	1.621161

61

dI1 – B3LYP/aug–CC–PVTZ

C	-2.586009	2.630183	1.437857
O	-2.618100	1.635659	0.367836
C	-2.871126	2.282706	-0.912807
C	-3.405274	3.667319	-0.560462
C	-2.648324	3.987399	0.738849
Mg	-1.615684	-0.237175	0.569706
O	-3.465922	-1.043798	-0.249139
C	-3.747275	-1.771097	-1.481216
C	-5.101686	-2.436170	-1.255251
C	-5.812068	-1.431208	-0.335671
C	-4.675294	-0.943805	0.560962
Cl	-0.312101	-1.599485	-1.027452
Mg	1.947321	-0.189471	-0.866612
O	2.768459	-1.944480	-0.054686
C	2.723297	-2.699960	0.917656
C	2.349868	0.106046	-2.962559
O	3.800848	0.680361	-0.049437
C	5.062100	0.154328	-0.560580
C	5.989469	1.360124	-0.700705
C	5.471148	2.305291	0.394026
C	3.963760	2.077592	0.331035
Cl	0.606741	1.322299	0.539201
C	-1.792582	-0.819713	2.635185
H	2.642214	-0.837896	-3.462178
H	3.162954	0.827178	-3.170745
H	1.463143	0.473590	-3.512089
H	5.442389	-0.574469	0.168293
H	4.854916	-0.353911	-1.508389
H	5.871611	1.820550	-1.690703
H	7.042578	1.084387	-0.570230
H	5.734199	3.354813	0.216959
H	5.864199	2.009823	1.376545
H	3.487996	2.705383	-0.435390
H	3.445277	2.223031	1.283312
H	-1.880865	-1.916004	2.757625
H	-0.937806	-0.508250	3.262584
H	-2.694386	-0.388473	3.109615
H	-3.781168	-1.047302	-2.308339
H	-2.923233	-2.469136	-1.649849
H	-4.974034	-3.400856	-0.745635
H	-5.634796	-2.609537	-2.197363
H	-6.626486	-1.878866	0.245812
H	-6.223664	-0.599371	-0.923183
H	-4.542461	-1.580784	1.444994
H	-4.778112	0.098489	0.880673
H	-1.923761	2.339434	-1.466540
H	-3.579949	1.654243	-1.462837
H	-4.487716	3.626772	-0.378437
H	-3.214505	4.394038	-1.358446
H	-3.153972	4.740011	1.354774
H	-1.636035	4.346131	0.511994
H	-3.459333	2.452350	2.079519
H	-1.669767	2.474665	2.015715
C	3.501940	-3.983883	0.905566
H	4.007018	-4.140771	1.867866
H	2.788706	-4.813805	0.782313
H	4.219456	-3.998120	0.080103
C	1.913485	-2.381603	2.140202
H	1.441042	-3.288519	2.537941
H	2.607794	-2.018799	2.915010
H	1.163187	-1.611323	1.942747

61
dP1 − B3LYP/aug−CC−PVTZ

C	3.554026	−1.463337	0.682770
O	2.350178	−1.178830	−0.095485
C	2.335024	−1.975012	−1.317231
C	3.768907	−2.466863	−1.483532
C	4.229436	−2.636681	−0.026804
Mg	0.816474	0.170846	0.480136
O	2.459633	1.609136	0.177950
C	2.516998	2.905349	0.850728
C	3.621423	3.678372	0.138681
C	3.506788	3.157881	−1.301627
C	3.176260	1.681549	−1.091263
Cl	−0.308155	1.505259	−1.381896
Mg	−1.834173	−0.419147	−1.273885
C	−2.562527	−1.387420	−3.043589
O	−3.473712	0.535060	−0.371681
C	−4.827669	0.455949	−0.914918
C	−5.709577	1.165504	0.108089
C	−4.761071	2.230228	0.682710
C	−3.426305	1.491981	0.734032
H	−2.893832	−0.668699	−3.815834
H	−3.428404	−2.046646	−2.846421
H	−1.798136	−2.024775	−3.525836
H	−4.834988	0.963705	−1.888997
H	−5.069170	−0.602054	−1.057378
H	−6.024051	0.466184	0.894470
H	−6.606209	1.593592	−0.354382
H	−5.066221	2.586847	1.672970
H	−4.697367	3.092938	0.006604
H	−3.303117	0.923018	1.664406
H	−2.552602	2.132282	0.579672
H	1.539581	3.394573	0.739669
H	2.704906	2.715049	1.910058
H	4.603187	3.428041	0.563884
H	3.476193	4.762160	0.217644
H	4.424492	3.291506	−1.885492
H	2.683755	3.660991	−1.825444
H	4.084098	1.070520	−1.000430
H	2.530279	1.266623	−1.871130
H	1.627010	−2.802349	−1.182893
H	1.984933	−1.330809	−2.130042
H	4.376864	−1.710720	−1.997406
H	3.815094	−3.398313	−2.058661
H	5.318934	−2.604825	0.084754
H	3.869048	−3.590662	0.379500
H	4.174897	−0.559121	0.682665
H	3.245330	−1.687182	1.709824
Cl	0.603146	0.837740	2.764760
O	−0.687531	−1.147229	0.183695
C	−1.053079	−2.313518	0.943123
C	−2.213879	−1.963105	1.891618
H	−3.093351	−1.629611	1.324384
H	−2.508337	−2.837160	2.489301
H	−1.912039	−1.159976	2.575416
C	−1.490960	−3.430719	−0.021659
H	−0.674648	−3.685823	−0.711062
H	−1.769870	−4.337472	0.532786
H	−2.357826	−3.123189	−0.621911
C	0.139672	−2.814262	1.774768
H	−0.150319	−3.696560	2.361511
H	0.975254	−3.101368	1.125911
H	0.478939	−2.039359	2.473454
